# COPD overdiagnosis in primary care: a UK observational study of consistency of airflow obstruction

**DOI:** 10.1038/s41533-019-0145-7

**Published:** 2019-08-15

**Authors:** Lynn Josephs, David Culliford, Matthew Johnson, Mike Thomas

**Affiliations:** 1 Department of Primary Care & Population Sciences, Faculty of Medicine, University of Southampton, Southampton, UK; 2 NIHR Collaboration for Leadership in Applied Health Research and Care for Wessex, University of Southampton, Southampton, UK; 3 NIHR Collaboration for Leadership in Applied Health Research and Care for Wessex Data Science Hub, Faculty of Health Sciences, University of Southampton, Southampton, UK; 4 NIHR Respiratory Biomedical Research Unit, Southampton General Hospital, Southampton, UK

**Keywords:** Medical research, Chronic obstructive pulmonary disease

## Abstract

Chronic obstructive pulmonary disease (COPD) is heterogeneous, but persistent airflow obstruction (AFO) is fundamental to diagnosis. We studied AFO consistency from initial diagnosis and explored factors associated with absent or inconsistent AFO. This was a retrospective observational study using patient-anonymised routine individual data in Care and Health Information Analytics (CHIA) database. Identifying a prevalent COPD cohort based on diagnostic codes in primary care records, we used serial ratios of forced expiratory volume in 1 s to forced vital capacity (FEV1/FVC%) from time of initial COPD diagnosis to assign patients to one of three AFO categories, according to whether all (persistent), some (variable) or none (absent) were <70%. We described respiratory prescriptions over 3 years (2011–2013) and used multivariable logistic regression to estimate odds of absent or variable AFO and potential predictors. We identified 14,378 patients with diagnosed COPD (mean ± SD age 68.8 ± 10.7 years), median (IQR) COPD duration of 60 (25,103) months. FEV1/FVC% was recorded in 12,491 (86.9%) patients: median (IQR) 5 (3, 7) measurements. Six thousand five hundred and fifty (52.4%) had persistent AFO, 4507 (36.1%) variable AFO and 1434 (11.5%) absent AFO. Being female, never smoking, having higher BMI or more comorbidities significantly predicted absent and variable AFO. Despite absent AFO, 57% received long-acting bronchodilators and 60% inhaled corticosteroids (50% and 49%, respectively, in those without asthma). In all, 13.1% of patients diagnosed with COPD had unrecorded FEV1/FVC%; 11.5% had absent AFO on repeated measurements, yet many received inhalers likely to be ineffective. Such prescribing is not evidence based and the true cause of symptoms may have been missed.

## Introduction

Over a million people have been diagnosed with chronic obstructive pulmonary disease (COPD) in the UK and it is likely that twice as many remain undiagnosed, yet overdiagnosis may also be a problem.^1,2^ While spirometry is fundamental to diagnosis,^3^ the National COPD Audit Programme^4^ reported in 2016 that, on cross-sectional analysis of general practitioner (GP)-held medical records assessing the most recently recorded spirometry, one quarter of values were not consistent with COPD. This has important clinical implications, if inappropriate treatments are being prescribed and the real causes of symptoms are being missed.

COPD is characterised by airflow obstruction (AFO) not fully reversible, not changing markedly over several months and usually progressive.^5^ AFO is due to a combination of airway and parenchymal damage resulting from chronic inflammation, in the West primarily as a result of smoking, with other factors, especially occupational exposure, contributing to the development of COPD.^3^ Dyspnoea, a prominent symptom, is multidimensional^6^ and may be misattributed to COPD in patients who have risk factors but lack AFO.

In this observational study using routine clinical data from primary care electronic patient records, our objective was to study the consistency of confirmatory spirometry for COPD diagnosis and explore factors associated with the absence of AFO. In the UK, GPs are incentivised to measure and record spirometry annually in COPD, so most patients have repeat readings recorded. After identifying a living cohort of patients with diagnosed COPD, we studied serial spirometry records from the time of initial diagnosis to look for evidence and consistency of AFO and examine characteristics, comorbidities and respiratory medication in those in whom AFO was consistent, inconsistent or entirely absent. We looked for factors that were predictive of absent AFO (suggesting overdiagnosis of COPD) and factors that might identify inconsistent AFO (raising the possibility of COPD misdiagnosis based on a single spirometry test).

## Results

We identified 14,378 patients with a primary care diagnosis of COPD with continuous data and alive at 31 December 2013: 53.1% male, mean ± SD age 68.8 ± 10.7 years. Median (interquartile range (IQR)) time from original COPD diagnosis to the study baseline (1 January 2011) was 60 (25, 103) months.

FEV1/FVC% was recorded in 12,491 patients (86.9%), with median (IQR) of 5 (3, 7) measurements from original COPD diagnosis to the end of 2013: 6550 (52.4%) showed persistent AFO, 4507 (36.1%) had FEV1/FVC% above and below the 70% threshold (variable AFO) and 1434 (11.5%) had no evidence of AFO on any measurement.

Table 1 summarises demographic and clinical characteristics for the total cohort (14,378), for those patients with known FEV1/FVC% (12,491) and for the 1887 (13.1%) with missing FEV1/FVC%. Patients with absent AFO were more often women and had a higher mean body mass index (BMI). Normal BMI (≥18.5 and <25 kg/m^2^) predominated in patients with persistent AFO, but overweight patients (≥25 and <30 kg/m^2^) predominated in those with variable and absent AFO (Supplementary Fig. 1). Median Medical Research Council (MRC) breathlessness score was similar in all the AFO groups; there was a greater proportion of patients scoring 1 in those without AFO (26.7%) compared to 22.3% and 21.2% in those with variable and persistent AFO, respectively, but the distribution of more breathless patients was similar across all AFO categories (Supplementary Fig. 2).

**Table 1:** Demographic and clinical characteristics by consistency of airflow obstruction

	COPD cohort (*n* = 14,378)						
	Total cohort (*n* = 14,378)	Consistency of recorded airflow obstruction from COPD diagnosis to end of study (*n* = 12,491, 86.9%)				Missing (no FEV_1_/FVC ratios)^a^ (*n* = 1887, 13.1%)	
		Persistent (all FEV_1_/FVC ratios <70%)^a^ (*n* = 6550, 52.4%)	Variable (some FEV_1_/FVC ratios <70%)^a^ (*n* = 4507, 36.1%)	Absent (all FEV_1_/FVC ratios ≥70%)^a^ (*n* = 1434, 11.5%)	*p* value	Subjects with NO spirometry (*n* = 1080, 57.2%)	Subjects with SOME spirometry (*n* = 807, 42.8%)
Gender, male, *n* (%)	7639 (53.1)	3785 (57.8)	2252 (50.0)	645 (45.0)	<0.001	516 (47.8)	441 (54.6)
Age, years, mean (SD)	68.8 (10.7)	68.6 (10.1)	69.9 (10.2)	67.2 (11.5)	<0.001	68.5 (14.0)	68.1 (11.2)
IMD, rank decile, median (IQR)	5 (2, 8)	5 (2, 8)	5 (2, 7)	5 (1, 7)	0.024	5 (2, 8)	5 (3, 7)
BMI, kg/m^2^, mean (SD)	27.4 (5.9)	26.5 (5.6)	28.2 (5.8)	29.5 (6.5)	<0.001	26.8 (6.1)	27.0 (6.0)
Smoking status, *n* (%)					<0.001		
Active smoker	5147 (39.4)	2583 (41.7)	1567 (35.6)	467 (35.8)		197 (43.9)	333 (46.4)
Ex-smoker	7658 (58.6)	3540 (57.1)	2746 (62.5)	783 (60.0)		222 (49.4)	367 (51.1)
Never smoker	262 (2.0)	77 (1.2)	83 (1.9)	54 (4.1)		30 (6.7)	18 (2.5)
FEV1, %predicted, mean (SD)	61.8 (19.6)	55.2 (17.7)	65.9 (18.2)	79.3 (19.0)	<0.001	n/a	62.5 (20.0)
FEV1, litres, mean (SD)	1.56 (0.62)	1.43 (0.57)	1.63 (0.61)	1.98 (0.69)	<0.001	n/a	1.56 (0.62)
FVC, litres, mean (SD)	2.66 (0.93)	2.78 (0.94)	2.53 (0.92)	2.57 (0.89)	<0.001	n/a	2.57 (1.15)
FEV1/FVC%, mean (SD)	60.2 (14.7)	51.4 (10.4)	65.9 (12.4)	80.5 (7.6)	<0.001	n/a	n/a
Number of FEV1/FVC% measurements, median (IQR)	5 (3, 7)	4 (2, 7)	6 (4, 8)	3 (1, 5)	<0.001	n/a	n/a
MRC dyspnoea, median (IQR)	2 (2, 3)	2 (2, 3)	2 (2, 3)	2 (1, 3)	0.006	3 (2, 4)	2 (1, 3)
Months since first COPD diagnosis, median (IQR)	60 (25,103)	57 (24,101)	65 (32, 108)	51 (20, 96)	<0.001	57 (24, 113)	58.5 (22,104)
Number of comorbidities, mean (SD)	2.5 (1.7)	2.3 (1.6)	2.8 (1.8)	2.9 (1.8)	<0.001	2.2 (1.6)	2.3 (1.7)

Missing data for baseline characteristics, *n* (%): IMD, 61 (0.4); BMI, 1720 (12.0); smoking status, 1311 (9.1); MRC dyspnoea score, 3052 (21.2)

*IMD* index of multiple deprivation (a weighted standardised measure of socio-economic status), *BMI* body mass index, *FEV*_*1*_ forced expiratory volume in 1 s, *FVC*, forced vital capacity, *MRC* Medical Research Council, *COPD* chronic obstructive pulmonary disease, *IQR* interquartile range, *n/a*, not applicable

^a^Test for difference depends on variable type/summary measures stated: ANOVA (for mean, SD), Kruskal–Wallis (for median, IQR) and Chi-squared test (for *n*, %)

Multivariable logistic regression (absent AFO versus persistent/variable AFO combined) showed that being older and male significantly reduced the odds of having absent AFO but that being an ex-smoker, never smoker, having a higher BMI or more comorbidities significantly increased the odds (Table 2). Never smokers (odds ratio (OR) 3.19, 95% confidence interval (CI): 2.25–4.54, *p* < 0.001) and those with a BMI ≥ 35 (OR 2.53, 95% CI: 2.07–3.09, *p* < 0.001) had the highest chance of having absent AFO.

**Table 2:** Predictors of absent AFO versus variable or persistent AFO^a^

	Odds ratio (OR)^b^	95% confidence interval	*p* value
Age (per year)	0.974	(0.968, 0.980)	<0.001
Gender			
Male	0.726	(0.643, 0.820)	<0.001
Smoking status			
Current smoker (reference)	1.000		
Ex-smoker	1.196	(1.044, 1.370)	0.010
Never smoker	3.192	(2.247, 4.535)	<0.001
Body mass index, kg/m^2^			
≥18.5 and <25 (reference)	1.000		
<18.5	1.065	(0.713, 1.592)	0.757
≥25 and <30	1.560	(1.323, 1.839)	<0.001
≥30 and <35	2.035	(1.701, 2.434)	<0.001
≥35	2.529	(2.071, 3.088)	<0.001
Number of diagnosed comorbidities (for each)	1.136	(1.097, 1.177)	<0.001

*AFO* airflow obstruction

^a^Using multivariable logistic regression where the outcome is the odds of absent AFO versus persistent/variable AFO, and all values of predictor variables are as at baseline

^b^Estimated for *N* = 11,331 subjects having values observed for all the variables shown in this table, all of which were used in this multivariable regression model

Table 3 shows the prevalence of comorbidities and statistical significance of any difference between prevalence in the three AFO categories. Asthma codes recorded between 2011 and 2013 showed no difference, while “ever” asthma was significantly increased in patients with variable AFO. All other diseases with a statistically significant difference were least prevalent in patients with persistent AFO; only lung cancer showed a trend towards an increase in this group. Multivariable logistic regression (absent AFO versus persistent/variable AFO combined), using individual comorbidities (rather than the number of comorbidities) in the prediction model, together with age, gender, smoking status and BMI, showed significantly increased odds of absent AFO in patients with recorded anxiety/depression, cerebrovascular disease, chronic kidney disease, diabetes mellitus, gastro-oesophageal reflux, hyperlipidaemia, pulmonary fibrosis or rhinosinusitis (Supplementary Table 1).

**Table 3:** Comorbidities by consistency of airflow obstruction

Individual comorbidities (*n*, %)	Cohort (*n* = 14,378)					
	Consistency of recorded airflow obstruction prior to study commencement (*n* = 12,491, 86.9%)				Missing FEV1/FVC% (no FEV_1_/FVC ratios available) (*n* = 1887, 13.1%)	
	Persistent (all FEV_1_/FVC ratios < 70%)^a^ (*n* = 6550, 52.4%)	Variable (some FEV_1_/FVC ratios < 70%)^a^ (*n* = 4507, 36.1%)	Absent (all FEV_1_/FVC ratios ≥ 70%)^a^ (*n* = 1434, 11.5%)	*p* value	Subjects with NO spirometry (*n* = 1080, 57.2%))	Subjects with SOME spirometry (*n* = 807, 42.8%)
Anxiety or depression	2341 (35.7)	1865 (41.4)	676 (47.1)	<0.001	396 (36.7)	307 (38.0)
Asthma codes ever	3337 (50.9)	2452 (54.4)	728 (50.8)	0.001	398 (36.9)	347 (43.0)
Asthma codes 2011–2013	1842 (28.1)	1331 (29.5)	425 (29.6)	0.209	163 (15.1)	173 (21.4)
Bronchiectasis	226 (3.5)	202 (4.5)	72 (5.0)	0.003	38 (3.5)	25 (3.1)
Cerebrovascular disease	445 (6.8)	420 (9.3)	137 (9.6)	<0.001	111 (10.3)	63 (7.8)
CKD	763 (11.6)	740 (16.4)	244 (17.0)	<0.001	117 (10.8)	105 (13.0)
Connective tissue disease	146 (2.2)	115 (2.6)	41 (2.9)	0.284	24 (2.2)	15 (1.9)
Dementia	71 (1.1)	59 (1.3)	24 (1.7)	0.158	55 (5.1)	9 (1.1)
Diabetes	732 (11.2)	688 (15.3)	266 (18.5)	<0.001	150 (13.9)	114 (14.1)
GORD	617 (9.4)	613 (13.6)	209 (14.6)	<0.001	94 (8.7)	76 (9.4)
Heart failure	275 (4.2)	258 (5.7)	89 (6.2)	<0.001	53 (4.9)	40 (5.0)
Hyperlipidaemia	1089 (16.6)	932 (20.7)	341 (23.8)	<0.001	133 (12.3)	115 (14.3)
Hypertension	2511 (38.3)	2006 (44.5)	614 (42.8)	<0.001	386 (35.7)	336 (41.6)
IHD	1009 (15.4)	924 (20.5)	276 (19.2)	<0.001	181 (16.8)	151 (18.7)
Lung cancer	39 (0.6)	20 (0.4)	<6 (<0.5)	0.070	<6 (<0.6)	6 (0.7)
OSA	58 (0.9)	59 (1.3)	24 (1.7)	0.014	7 (0.6)	<6 (<0.7)
Osteoporosis	375 (5.7)	300 (6.7)	86 (6.0)	0.131	80 (7.4)	44 (5.5)
PVD	328 (5.0)	252 (5.6)	60 (4.2)	0.090	54 (5.0)	36 (4.5)
PF	57 (0.9)	67 (1.5)	28 (2.0)	<0.001	7 (0.6)	8 (1.0)
Rhinosinusitis	949 (14.5)	772 (17.1)	258 (18.0)	<0.001	113 (10.5)	119 (14.7)

*CKD* chronic kidney disease, *GORD*, gastro-oesophageal reflux disease, *IHD* ischaemic heart disease, *FEV*_*1*_ forced expiratory volume in 1 s, *FVC* forced vital capacity, *OSA* obstructive sleep apnoea, *PVD* peripheral vascular disease, *PF* pulmonary fibrosis

^a^Test for difference depends on variable type/summary measures stated: ANOVA (for mean, SD), Kruskal–Wallis (for median, IQR) and Chi-squared test (for *n*, %)

When patients with variable AFO were compared with those with persistent AFO, multivariable logistic regression showed that being male or underweight (BMI < 18.5) significantly reduced the odds of having variable AFO but that being older, a never smoker, having a higher BMI or more comorbidities significantly increased the odds (Table 4). A BMI ≥ 35 (OR 2.13, 95% CI: 1.83–2.47, *p* < 0.001) was the strongest predictor of variable AFO. When an asthma “ever” diagnosis was adjusted for, as a separate covariate in the multivariable model, asthma “ever” was not a significant predictor of variable AFO (OR 1.003, 95% CI: 0.924–1.088, *p* = 0.946).

**Table 4:** Predictors of variable AFO versus persistent AFO^a^

	Odds ratio (OR)^b^	95% confidence interval	*p* value
Age (per year)	1.007	(1.003, 1.011)	0.002
Gender			
Male	0.725	(0.669, 0.787)	<0.001
Smoking status			
Current smoker (reference)	1.000		
Ex-smoker	1.082	(0.990, 1.183)	0.083
Never smoker	1.455	(1.048, 2.020)	0.025
Body mass index, kg/m^2^			
≥18.5 and <25 (reference)	1.000		
<18.5	0.739	(0.580, 0.941)	0.014
≥25 and <30	1.425	(1.291, 1.573)	<0.001
≥30 and <35	1.794	(1.594, 2.018)	<0.001
≥35	2.126	(1.830, 2.470)	<0.001
Number of diagnosed comorbidities (for each)	1.116	(1.089, 1.144)	<0.001

*AFO* airflow obstruction

^a^Using multivariable logistic regression where the outcome is the odds of variable AFO versus persistent AFO, and all values of predictor variables are as at baseline

^b^Estimated for *N* = 10,105 subjects having values observed for all the variables shown in this table, all of which were used in this multivariable regression model

Table 5 summarises inhaled medication over the study period. It shows the number of patients in each AFO category receiving each drug class and a summary measure of prescribing (number of 3-month periods in which ≥1 prescription was received, range 1–12). Patients with absent AFO were prescribed less medication, but 73.6% received short-acting bronchodilators, 57.3% long-acting bronchodilators, 60.1% inhaled corticosteroids (ICS) and 45.8% ICßS and long-aßcting beta_2_ agonists (LABA); only 20.2% received no inhaled medication.

**Table 5:** Inhaled treatment during the 3-year study period, by categories of AFO

	Persistent AFO (*n* = 6550)	Variable AFO (*n* = 4507)	Absent AFO (*n* = 1434)	No spirometry (*n* = 1080)	Incomplete spirometry (*n* = 807)
Short-acting bronchodilators *n* (%), median [IQR]^a^	5410 (82.6) 8 [4, 10]	3724 (82.6) 7 [4, 10]	1055 (73.6) 6 [3, 9]	368 (34.1) 5 [2, 9]	628 (77.8) 8 [4, 10]
Long-acting bronchodilators *n* (%), median [IQR]^a^	5017 (76.6) 10 [7, 10]	3329 (73.9) 9 [6, 10]	822 (57.3) 8 [5, 10]	277 (25.6) 5 [2, 9]	561 (69.5) 9 [6, 10]
Inhaled corticosteroids *n* (%), median [IQR]^a^	4642 (70.9) 11 [7, 12]	3168 (70.3) 10 [6, 12]	862 (60.1) 9 [5, 12]	300 (27.8) 6 [2, 10]	503 (62.3) 10 [7, 12]
ICS/LABA^b^ *n* (%), median [IQR]^a^	4117 (62.9) 9 [6, 10]	2703 (60.0) 8 [5, 10]	657 (45.8) 7 [4, 10]	230 (21.3) 5 [2, 8]	438 (54.3) 9 [5, 10]
None of the above treatments, *n* (%)	857 (13.1)	540 (12.0)	290 (20.2)	672 (62.2)	145 (18.0)

Short-acting bronchodilators comprise short-acting beta_2_ agonists (SABA) and short-acting antimuscarinic bronchodilators (SAMA). Long-acting bronchodilators comprise long-acting beta_2_ agonists (LABA) and long-acting antimuscarinic bronchodilators (LAMA)

*AFO* airflow obstruction, *ICS* inhaled corticosteroids, *IQR* interquartile range

^a^Number of patients receiving each class of medication and the number of 3-month periods (maximum of 12) in which ≥1 prescription was received

^b^Includes patients receiving ICS/LABA in fixed combination inhalers or separately

Having observed the high level of inhaler prescribing in patients without AFO, a secondary subset analysis was performed on patients without asthma codes during the study period (*n* = 10,444). Table 6 shows prescribing in these patients: of 1009 (9.7%) with diagnosed COPD, no recorded asthma and absent AFO, 49.7% received long-acting bronchodilators, 49.0% ICS and 36.2% ICS and LABA.

**Table 6:** Inhaled treatment for patients without in-study asthma codes during the 3-year study period, by categories of AFO

	Persistent AFO (*n* = 4708)	Variable AFO (*n* = 3176)	Absent AFO (*n* = 1009)	No spirometry (*n* = 917)	Incomplete spirometry (*n* = 634)
Short-acting bronchodilators *n* (%), median [IQR]^a^	3654 (77.6) 7 [4, 10]	2481 (78.1) 7 [3, 10]	668 (66.2) 5 [2, 8]	226 (24.6) 4 [1, 7]	463 (73.6) 7 [4, 10]
Long-acting bronchodilators *n* (%), median [IQR]^a^	3366 (71.5) 10 [7, 10]	2178 (68.6) 9 [6, 10]	501 (49.7) 8 [5, 10]	163 (17.8) 4 [1, 7]	420 (66.2) 9 [6, 10]
Inhaled corticosteroids *n* (%), median [IQR]^a^	2955 (62.8) 10 [7, 12]	1972 (62.1) 10 [6, 12]	494 (49.0) 8 [4, 11]	164 (17.9) 4 [2, 9]	350 (55.2) 10 [6, 12]
ICS/LABA^b^ *n* (%), median [IQR]^a^	2643 (56.1) 8 [5, 10]	1684 (53.0) 8 [5, 10]	365 (36.2) 7 [4, 10]	124 (13.5) 3.5 [1.25, 7]	313 (49.4) 8 [5, 10]
None of the above treatments, *n* (%)	836 (17.8)	528 (16.6)	281 (27.9)	658 (71.8)	142 (22.4)

Short-acting bronchodilators comprise short-acting beta_2_ agonists (SABA) and short-acting antimuscarinic bronchodilators (SAMA). Long-acting bronchodilators comprise long-acting beta_2_ agonists (LABA) and long-acting antimuscarinic bronchodilators (LAMA)

*AFO* airflow obstruction, *ICS* inhaled corticosteroids, *IQR* interquartile range

^a^Number of patients receiving each class of medication and the number of 3-month periods (maximum of 12) in which ≥1 prescription was received

^b^Includes patients receiving ICS/LABA in fixed combination inhalers or separately

There were 52 patients (0.4% of the cohort) whose COPD diagnosis was defined only by chronic bronchitis codes. Of these, FEV1/FVC% was recorded in 25 patients (48.1%): <6 had persistent AFO, 8 had variable AFO, and 13 had absent AFO. In 225 patients (1.6% of the cohort), COPD diagnosis was defined only by codes for acute exacerbation of COPD. Of these, AFO could be categorised in 109 patients (48.4%): 37 had persistent AFO, 25 had variable AFO, and 47 had absent AFO.

## Discussion

Our objectives were to study the consistency of AFO over time in a large and representative clinically defined UK primary care COPD cohort, using serial routine spirometry from the time of first diagnosis until the end of 2013, and to explore factors associated with the absence of confirmed AFO (suggesting overdiagnosis of COPD) and factors that might identify inconsistent AFO (raising the possibility of COPD misdiagnosis based on a single spirometry test).

In 1887 patients (13.1% of this large cohort), spirometry values were missing from the records: either completely absent or inadequate to calculate a FEV1/FVC ratio. In the remaining 12,491 patients with a COPD diagnosis and recorded FEV1/FVC ratios, 11.5% had no evidence of obstructive spirometry on any measurement of FEV1/FVC% from the time of diagnosis, so are highly likely to be misdiagnosed. AFO was variable in 36.1%, raising important questions about the validity of the diagnosis and/or the quality of spirometry. In only 52.4% did persistent AFO fully support the diagnosis. Being younger, female, a non-smoker or having a higher BMI or more comorbidities significantly increased the odds of absent AFO. Being female, a never smoker, having a higher BMI or more comorbidities were also predictors of variable AFO. Most patients without AFO were receiving inhaler treatments that did not appear to be explained by a concomitant diagnosis of asthma: after excluding patients with current asthma codes, 49.7% of patients with absent AFO were receiving long-acting bronchodilators and 49.0% ICS.

Other cross-sectional studies have suggested that overdiagnosis of COPD is common.^1,2,4,7^ Newly available international data, from the Burden of Obstructive Lung Disease (BOLD) study in 23 population samples across 20 countries, provide evidence of overdiagnosis and over treatment.^8^ Between 2003 and 2012, quality-assured spirometry was performed in 919 patients reporting a previous medical diagnosis of COPD; un-obstructive spirometry (a false positive COPD diagnosis) was found in more than half the study population (in 61.9% when the FEV1/FVC ratio boundary was defined by the lower limit of normal (LLN) or 55.3% with a fixed 70% cut-off). However, our study offers the novelty of serial measurements over a prolonged period of time, allowing us to categorise patients with spirometry readings as having persistent, variable or absent AFO from the time of initial COPD diagnosis. This strengthens the likelihood of genuine non-obstruction in those in whom AFO was always absent and, in other patients, is a measure of inconsistency of AFO since diagnosis. A recent study by Shermer et al.^9^ in symptomatic smokers and ex-smokers showed shifts in diagnostic categories between obstruction and non-obstruction following annual spirometry over 2 years, questioning the validity of a single measurement to diagnose COPD. A Latin American population survey of adults aged >40 years showed inconsistent AFO in repeat tests 5–9 years later, especially in FEV1/FVC values closest to the cut-off values.^10^

Others have also highlighted inappropriate prescribing in the absence of obstructive spirometry. Spyratos et al.^11^ studied ex-smokers and current smokers without asthma and found 9.6% overdiagnosis of COPD, of whom 35% were receiving inhalers in the past year. In the recently available BOLD Study data, in subjects with diagnosed COPD but un-obstructive spirometry, current use of respiratory medication was reported in 45.7%, 34.4% after excluding subjects with reported asthma.^8^ Our subgroup analysis provides similar evidence that inhaler prescribing was for a diagnosis of COPD and not explained by concurrent asthma.

We found that BMI was higher in those without AFO, as others have reported,^1,12,13^ with a preponderance of overweight and obese patients in this group. Obesity has multiple effects on spirometry^14^ and may be associated with dyspnoea that may be incorrectly attributed to COPD. Obese people cannot fully exhale from a resting respiratory position and this reduces FVC (and slow vital capacity) without reducing FEV1, so the FEV1/FVC ratio is increased. More severe obesity leads to fat deposition around the rib cage, causing chest wall restriction, which lowers the inspiratory capacity, affecting the VC and the FEV1 to a similar degree, so not affecting the FEV1/FVC ratio.

We found that cardiovascular and psychological comorbidities were more common in those without AFO, again raising the possibility of misattribution of symptoms. Indeed, most comorbidities were more common in those without AFO, with 13 of the 19 comorbidities studied being significantly more prevalent in this group than in those with persistent AFO. After adjusting for potential confounders (age, gender, smoking status, BMI and other comorbidities), 8 of these 13 chronic diseases were independent predictors of an increased odds of absent AFO. It seems plausible that the reporting of symptoms compatible with COPD but caused by other conditions in patients with typical COPD demography (particularly when accompanied by suggestive exposures such as smoking) may sometimes result in an inappropriate COPD diagnosis, even when spirometry readings are outside diagnostic limits. Quint et al.^15^ have shown that diagnostic accuracy of COPD decreased for all Read code algorithms when asthma or cardiovascular disease were present. The BOLD Study data also showed that COPD overdiagnosis was more common in subjects with comorbid asthma or heart disease.^8^

In the UK, COPD diagnosis is generally made by clinicians in primary care (GPs and nurses), with nurses usually performing the spirometry and playing a central role in routine care. National Institute for Health and Clinical Excellence (NICE) guidelines^5^ and “Quality and Outcome Framework” (QOF) (http://www.hscic.gov.uk/qof) standards require that diagnosis should be based on post-bronchodilator spirometry. QOF is an incentive scheme, in use since 2004, that financially rewards practices for delivering good quality care. Quality-assured spirometry is the gold standard for COPD diagnosis, but the test requires adequate training in both performance and interpretation. Just as patients without AFO can be misdiagnosed with COPD, a correct clinical diagnosis of COPD might be overlooked by failure to demonstrate AFO. It is acknowledged that spirometry performed in primary care may not be to national or international standards.^16^ A recent systematic scoping review of COPD misdiagnosis concluded that misdiagnosis was mainly attributable to inadequate spirometry and lack of correct interpretation by the health-care professional performing the assessment.^17^ Standardisation of spirometry training is evolving in line with national and international guidelines, under the stewardship of the Association for Respiratory Technology and Physiology (ARTP) (http://www.artp.org.uk/en/spirometry). However, at the time of this study there would have been less uniformity in training and in quality assurance. Technically poor spirometry is a possible explanation for failure to demonstrate AFO, as end-of-test criteria are commonly not met,^18^ resulting in underestimation of FVC and, consequently, falsely elevated FEV1/FVC ratios; mean FVC was lower in our patients with absent or inconsistent AFO by around 200 ml, when compared to patients with persistent obstruction. As we have no information on quality assurance of our recorded spirometry readings, it is possible that AFO might have been demonstrable with quality-assured spirometry or evident only during relaxed expiratory manoeuvres. It should be mentioned that not all spirometry values in our data set reflect spirometry performed in primary care, as some tests may have been performed in the hospital setting, with values entered into primary care records from information received in outpatient letters or discharge summaries.

We acknowledge other limitations and potential biases inherent in observational studies using routine clinical data, where motivation for data recording varies and codes are used inconsistently.^4,19^ To reduce misclassification, we identified our cohort using Read QOF codes that are used to define COPD for performance-related pay calculations. It is unlikely that COPD diagnosis codes would be used in primary care records before a firm clinical diagnosis had been made. We selected only diagnosis codes, avoiding symptom codes (likely to be used for undiagnosed patients presenting with symptoms such as cough or breathlessness), non-specific codes such as “smoker’s cough” or “suspected COPD” (codes that might be used prior to spirometry) or process-of-care codes such as “annual COPD review” (which might be used to follow up patients with other respiratory conditions). We believe that restricting our defining codes in this way increased the likelihood that a clinical diagnosis of COPD had been made in those patients included in our cohort. Although we cannot discount the possibility of a GP making a provisional COPD diagnosis, the fact that most patients received treatment indicates that this was a “firm” (although possibly mistaken) diagnosis. Earlier evidence from Quint et al.^15^ using the Clinical Practice Research Datalink, concluded that “the presence of a specific chronic obstructive pulmonary disease (COPD) Read code alone is sufficient to identify patients with COPD from electronic health records, with minimal precision lost by not including spirometry and medications in the algorithm”. Estimates of precision in that study were based on the *presence* of recorded spirometry and not on examination of actual spirometry values (when additional material permitted closer scrutiny of the GP diagnosis by respiratory physicians, any discordance was usually because lung function did not meet criteria for COPD).

There are other problems with diagnostic coding in primary care records. Codes often remain after a diagnosis has been called into question. When later non-obstructive spirometry does not support an earlier COPD diagnosis, the original code is unlikely to be deleted from the records. This may account for some of our patients with variable AFO, who may have had their diagnosis subsequently revised as asthma. Similarly, uncommon conditions such as bronchiectasis and pulmonary fibrosis (more prevalent in our patients with absent AFO) may have originally been misdiagnosed as COPD, with the incorrect code persisting in the records.

There are many reasons why spirometry data may be incorrect or missing in clinical databases: the test might be performed but results not or incorrectly coded; results may be in “free text” or only in hospital correspondence. Anticipating coding inconsistency, we used all FEV1/FVC codes to categorise our patients (not just those codes that imply post-bronchodilator measurements) though QOF and widespread use of electronic templates do encourage both correct coding and performance of post-bronchodilator spirometry. Had bronchodilators not actually been administered, this would tend to overestimate AFO in our cohort by including patients with fully or partially reversible AFO, especially likely in patients with asthma or concurrent asthma and COPD.

There are intrinsic problems in dichotomising continuous variables such as FEV1/FVC.^20^ Some patients may have a “true” FEV1/FVC% close to the 70% boundary and apparent variation is artefactual, due to inherent variation in test reliability. The more the tests are performed, the greater the likelihood of demonstrating variable AFO. In our study, the group with variable AFO did tend to have more measurements of FEV1/FVC% and a longer duration of COPD. Other studies have shown that effective treatment^21^ or stopping smoking^22^ may improve lung function sufficiently for FEV1/FVC% to cross the boundary. In some of our patients, treatment with bronchodilators or inhaled corticosteroids may have produced small improvements in lung function sufficient to alter the categorisation of those close to the 70% threshold. This might account for some of our patients with variable AFO but is unlikely to explain those with absent AFO, in whom spirometry at the time that the original COPD diagnosis was made should have confirmed AFO if the diagnosis was correct. At the study baseline, consistent electronic primary care records were available for 10–15 years, varying by practice. Although our study was designed to capture spirometry at the time of initial diagnosis, given the duration of COPD among our cohort, it is possible that there are a small number of patients for whom initial diagnosis and spirometry occurred before the advent of electronic records. While in this scenario a historic diagnosis that remains relevant to a patient’s current care, such as COPD, is often retrospectively recorded and backdated, historical events relating to past care, such as spirometry, are often absent.

Debate exists around defining AFO using a fixed threshold for FEV1/FVC, which tends to overdiagnose COPD in the elderly, due to normal age-related decline.^23,24^ There is evidence to support using the lower limit of normal (LLN), defined as the lower 5th percentile for predicted post-bronchodilator FEV1/FVC for the sex and age of the subject.^9,25^ However, international and UK guidelines still recommend the fixed ratio for diagnosis,^3,5^ and there is evidence of worse outcomes in subjects aged ≥65 years classified as normal using LLN but abnormal using the fixed ratio, with an increased risk of dying and of COPD-related hospitalisation.^26^ In our own study, there was insufficient data to reliably calculate LLN, but we would expect any “borderline” patients with inconsistency between “fixed ratio” and LLN criteria to have FEV1/FVC% close to the 70% cut-off. As FEV1/FVC% was low (mean ± SD 51.4 ± 10.4) in our “persistent AFO” category and high (mean ± SD 80.5 ± 7.6) in our “absent AFO” category, we do not believe many patients would be differently categorised had we used LLN. Indeed, the proportion of our cohort without evidence of AFO would likely be even greater, given our elderly population (mean age 68.8 years). Furthermore, as both mean FEV1/FVC% and FEV1% predicted were high in those without AFO, it is likely that most do not have chronic airways disease and have an entirely different cause for their symptoms, which may be unrecognised.

COPD diagnosis relies on clinical judgement based on symptoms, risk factors and evidence of post-bronchodilator AFO.^3^ It is a heterogeneous condition with different phenotypes.^27^ Chronic bronchitis and emphysema may occur without AFO, particularly in earlier stages.^28^ In a subgroup analysis within the recent BOLD study, unobstructed spirometry was observed less frequently when subjects reporting a diagnosis of chronic bronchitis or emphysema were excluded (37.7% of 220 subjects, compared to 61.9% of all 919 patients with a COPD diagnosis).^8^ Quint et al.^15^ concluded that some chronic bronchitis codes used by QOF to define COPD were insufficiently specific to reliably identify patients with COPD in electronic records. We considered whether chronic bronchitis might be prevalent among our patients with absent AFO and explain the diagnosis and treatment we observed, since 13 of our cohort-defining Read codes were for chronic bronchitis. However, only 0.4% of our patients overall and 0.9% of patients with absent AFO had their COPD diagnosis based solely on chronic bronchitis codes. We also examined 3 Read codes used to denote an acute exacerbation of COPD (AECOPD). These codes are excluded from the QOF COPD definition codes (to avoid duplicate payments to practices for patients experiencing multiple exacerbations). We included them because diagnosis may only be made after an acute exacerbation results in a hospital-based diagnosis,^29^ though not all patients who are hospitalised with AECOPD have their COPD diagnosis confirmed after recovery.^30,31^ In our cohort, only 1.6% were solely identified on the basis of AECOPD codes: 3.3% of those with absent AFO compared to 0.6% of those with either variable or persistent AFO.

Current and former smokers with normal spirometry may show clinical and radiological evidence of airway disease.^32,33^ From a research perspective, symptomatic smokers without AFO are an important group who require further study.^34,35^ In specialist care, such patients are often prescribed inhalers for pragmatic reasons but without an evidence base to support their use.^36^ However, in the absence of confirmatory trial evidence and a licence for the use of COPD-approved medication in this way, we feel it is inappropriate for such treatment to be commenced in primary care; in particular, a diagnosis of COPD should not be used to “justify” treatment in the absence of agreed AFO criteria being met. A further challenge is in differentiating COPD from asthma^37^: these disease labels may be incorrectly attributed,^38,39^ asthma may lead to intermittent or persistent AFO,^40^ and the two diseases may occur independently or overlap.^41^ In our cohort, codes for “ever” asthma were common across all AFO categories, though slightly higher (54.4%) in the group with variable AFO, as might be expected. However, multivariable analysis showed that “ever” asthma was not a predictor for variable AFO, after adjustment for confounding variables (age, gender, smoking status, BMI and number of other comorbidities). We did not have data for reversibility testing, which might have been useful for asthma diagnosis.

We believe our results are generalisable to a wider UK population. We have analysed routine primary care data recorded using a widely employed coding method (Read V2), so data quality issues are likely to be similar. Our cohort encompassed a broad socio-economic spectrum, as reflected in the range of index of multiple deprivation (IMD) scores (IQR 2–8, median 5) when these standardised measures of socio-economic status were ranked according to national deciles. Though reported ethnicity in Hampshire is largely White British (89% in the 2011 Census^42^), there is heterogeneity across the county, with urban areas having greater ethnic diversity than the national average.

In conclusion, patients without AFO require clinical reassessment, as they may be receiving inappropriate, potentially harmful^43^ and costly medications that may not benefit them, while the true cause of their symptoms may have been missed. Quality-assured post-bronchodilator spirometry is key to correct diagnosis and COPD should not be diagnosed without it. If absent AFO is confirmed, consideration should be given to the cautious withdrawal of inhaled treatment and to secondary care referral for those with persisting diagnostic uncertainty. Looking towards future research, evidence-based guidance is needed on effective management of those patients with symptoms and relevant exposures who may have early airways disease without airflow limitation.

## Methods

The study protocol is publically available.^44^ The study received ethical approval from the University of Southampton and governance approval from the Care and Health Information Exchange Information Governance Group (CHIE IGG).

We report our findings in line with STROBE^45^ and RECORD^46^ guidelines for observational studies using routinely collected health data (Supplementary Table 2, RECORD statement).

### Setting

This investigation was part of a retrospective observational cohort study of patients with a primary care diagnosis of COPD using individual patient-anonymised routinely collected clinical data held in Care and Health Information Analytics (CHIA), formerly Hampshire Health Record Analytics,^47^ an electronic NHS UK regional linked primary care database of around 1.4 million patients living in Hampshire, UK. A description of the database is given as Supplementary Information (Supplementary Methods). We have previously reported clinical outcomes (hospitalisation and mortality) in the original cohort over a 3-year period (2011–2013).^48^

### Participants

We identified a prevalent COPD cohort on 1 January 2011 on the basis of Read diagnostic codes for COPD being present in primary care records prior to that date. All patients aged >25 years with 3 years’ continuous data from 1 January 2011 and alive on 31 December 2013 were included in the study cohort. For each patient, we determined the date on which a COPD diagnosis was first recorded. Serial measurements of the ratio of FEV1/FVC% from the time of initial COPD diagnosis until 31 December 2013 were used to categorise individuals according to whether all, some or none were <70%, the level routinely used to define AFO in UK clinical practice.^3,5^

Patients were further characterised by demographic and clinical factors at baseline (1 January 2011): age, sex, smoking status, socio-economic deprivation (IMD), BMI, MRC breathlessness score, FEV1, FVC, FEV1/FVC%, FEV1 %predicted, and by the presence or absence of 19 comorbidities. Comorbidities were defined using Read codes for the following chronic conditions: anxiety/depression, asthma (“ever” or during the study period 2011–2013, defined as “current”), bronchiectasis, cerebrovascular disease, chronic kidney disease, connective tissue disease, dementia, diabetes mellitus, gastro-oesophageal reflux, heart failure, hyperlipidaemia, hypertension, ischaemic heart disease, lung cancer, obstructive sleep apnoea, osteoporosis, peripheral vascular disease, pulmonary fibrosis, and rhino-sinusitis. Respiratory medication was documented over 3 years, 2011–2013, categorised by drug class: short-acting beta_2_ agonists, short-acting antimuscarinic bronchodilators, LABA, long-acting antimuscarinic bronchodilators, and ICS.

Additional details and code lists for all variables are available as Supplementary Information (Supplementary Methods).

### Statistical methods

Details of data handling within CHIA (data access, composition and cleaning) are described in Supplementary Methods.

Using a data set containing serial measurements of FEV1/FVC% from 4 weeks before the date of first COPD code (assumed to reflect the date of a patient’s initial COPD diagnosis) until 31 December 2013, three patient groups were defined: patients with persistent AFO (all FEV1/FVC% <70%), variable AFO (some FEV1/FVC% <70%), and absent AFO (all FEV1/FVC ≥70%). In patients without Read-coded ratios for FEV1/FVC but with coded individual components (FEV1 and FVC values recorded on the same day), FEV1/FVC% was calculated. The remaining patients with missing FEV1/FVC% were categorised according to whether spirometry data were incomplete or totally absent.

Time since COPD diagnosis was estimated from the interval between first COPD code and the study baseline (1 January 2011).

Summary measures were used to describe patient characteristics: mean and standard deviation for all except IMD, MRC score, months from initial COPD diagnosis, and the number of FEV1/FVC% from initial diagnosis to the end of the study (31 December 2013), where median and IQR were used. Respiratory medication over 3 years (2011–2013) was analysed by drug class and summarised by ≥1 prescription in each 3-month period over 3 years, giving a range for this variable of 1–12.

Multivariable logistic regression was used to estimate associations between the odds of having absent AFO (versus persistent/variable AFO combined) and potential predictors (age, gender, smoking status, BMI and number of comorbidities). A sensitivity analysis used multivariable logistic regression to estimate associations between the odds of having absent AFO and individual comorbidities, adjusting for age, gender, smoking status and BMI.

Multivariable logistic regression was used to estimate associations between the odds of having variable AFO (versus persistent AFO) and potential predictors (age, gender, smoking status, BMI and number of comorbidities), with a sensitivity analysis in which an asthma “ever” diagnosis was a separate covariate in the multivariable model.

Two further sensitivity analyses were performed to explore diagnostic codes used to define the COPD cohort. We determined the number of patients in each AFO category who were in the COPD cohort by virtue of having only chronic bronchitis codes (codes that do not imply AFO) or only codes for acute exacerbation of COPD (codes defined in Supplementary Methods).

All parameter estimates are presented with 95% CIs. All tests were conducted as two sided, at the 5% significance level. Analyses were conducted using the statistical software packages SAS v9.4 (SAS Institute: Cary, NC), SPSS v22 (IBM Corp: Armonk, NY) and R v3.1 (R Core Team: Vienna, Austria).

### Reporting summary

Further information on research design is available in the Nature Research Reporting Summary linked to this article.

## Supplementary information

Supplementary Information

Reporting summary

## Data Availability

Individual-level data used for this study are held within the Care and Health Information Analytics (CHIA) safe haven environment and are not available to be shared. Readers are able to refer to our Supplementary material to see the rationale, method and clinical codes underpinning the data extraction phase of the study, so a similar study could be reproduced by researchers using data sourced from any similarly structured clinical database.

## Supplementary information

**Supplementary Information** accompanies the paper on the *npj Primary Care Respiratory Medicine* website (10.1038/s41533-019-0145-7).

## Code availability

No algorithms or custom program codes were written as part of the analysis, which were deemed central to the specific conclusions found. Only standard use of existing routines within software for data manipulation and statistical analysis was employed.
