# Supplementary material for: COPD overdiagnosis in primary care: a UK observational study of consistency of airflow obstruction
Source: NPJ Prim Care Respir Med. 2019 Aug 15;29:33. doi: 10.1038/s41533-019-0145-7 (PMC6695394; doi:10.1038/s41533-019-0145-7)
Supplement: Supplementary file 1 — Supplementary Information [file 41533_2019_145_MOESM1_ESM.pdf]

Supplementary Information

Supplementary Methods

The database

The Care and Health Information Exchange (CHIE), formerly Hampshire Health Record (HHR),[1] is an electronic shared care record for people living in Hampshire and the Isle of Wight, in the South of England. It provides a single source of linked routine health data for individual patients, collected in "real-time", linking data from GP practices and hospitals (and some data from social care). It can extract data from most GP computerised software systems. It is not part of the National Summary Care Record but rather is a local arrangement, storing data from a wide range of practices that have "opted in" to the data-sharing scheme.

The Care and Health Information Analytics (CHIA) database, formerly known as the Hampshire Health Record Analytical database (HHRA), is a separate electronic database created for research and analysis to support health improvement and planning. At the time this study was undertaken, the database included data from more than 140 practices across the county of Hampshire, covering about 1.4 million patients (approximately 75% of Hampshire patients and practices). It shares some of the health data contained within CHIE but in anonymised form to protect patients' identity. During the time of the study, linked data contained within HHRA included primary care data (coded clinical entries made during routine patient care) and secondary care data (in-patient, out-patient and Emergency Department (ED) Secondary Uses Service (SUS) data and radiology and pathology data). In order to preserve patients' anonymity, age is based on year of birth and index of multiple deprivation (IMD)[2] is assigned from Lower Layer Super Output Area (LSOA) codes rather than postcodes. Patients' anonymity is further protected by not reporting exact patient numbers when there are fewer than six in any population: in this situation, we report n<6. IMD is a weighted standardised measure of socio-economic status based on several factors (including income, health, living environment, education and employment). We have used the national IMD rank of Hampshire LSOAs and ordered them amongst all England LSOAs to sort into deciles.

CHIE and CHIA are hosted and maintained by NHS South, Central and West Commissioning Support Unit (CSU). The Governance body is the Care and Health Information Exchange Information Governance Group (CHIE IGG), which ensures the security and confidentiality of CHIE and CHIA and considers issues of data integration or data sharing.

Although not all Hampshire practices submit data to CHIA, missing practices are dispersed across the catchment area, with varying rural/urban classification, socioeconomic deprivation and patient composition. We are not aware of any systematic differences between missing practices and those whose data are represented.

Data handling

The investigators had access to anonymised records for all patients registered with practices that had "signed up" to CHIE and CHIA, with the exception of patients whose records were coded to indicate that they had specifically opted out of the process. Only one investigator (MJ) had direct involvement with the CSU that administers the database, and its governance body, CHIE IGG.

The composition of the database population changes slightly with time (because enrolling practices cease collaborating or join because of changes in practice software) so all analyses were performed on a single version of the data.

Basic data cleaning had already been performed within the NHS environment before data were imported into CHIE and CHIA. For this project, data cleaning involved excluding from the dataset duplicated records and assessments with null and zero values and values deemed incompatible with life. The following out-of-range (OOR) rules were created:

BMI: <10 or >70 Kg/m²

FEV1: <0.2 or >7.0 litres

FEV1 %predicted: <10 or >140%

FEV1/FVC %: <10 or >100%

We used FEV1 volumes to derive %predicted FEV1 values when the latter were missing, using standard prediction equations.[3]

We calculated FEV1/FVC% ratios where they were missing from patient records, but FEV1 and FVC values had been separately recorded on the same day (this occurred in 484 patients).

Read codes: methodology and code lists

Read codes are the standard clinical terminology system used in primary care in the UK since 1985. The Read code system comprises a detailed hierarchy of 5-character codes**,** which are grouped into chapters that describe diagnoses, symptoms, examination findings, investigations, administrative items, procedures and medication. Higher level "parent" codes give rise to lower level "child" codes with increasing definition specificity. Each code is linked to a descriptive Read term (or more than one synonymous term) allowing the practitioner to select the appropriate term (and therefore code) during routine patient care. At the time this project was undertaken, ninety percent of UK practices used Read Version 2 (V2) codes and these were used in our study.

The directory of Read codes, the "Read Browser" is administered by the UK Terminology centre "Technology Reference data Update Distribution Service" (TRUD),[4] part of the Health and Social Care Information Centre. The Browser contains over 86,000 V2 codes and receives twice-yearly updates (April and October) from TRUD, when new codes are added and a few redundant codes withdrawn. In order to ensure that we included any relevant new codes introduced during our cohort follow-up (2011-2013), we compiled lists that included codes from the October 2013 Read Browser. An Access database was developed to save the contents of the Read Browser (a flat file database) in a format that greatly facilitated searching Read codes and Read terms and creating code lists.

We identified our COPD cohort based solely on selected diagnostic Read codes from the Respiratory Disease chapter of the Read Browser. We did not include any "process of care codes" (codes for symptoms, signs, management and administration) nor did we select our cohort based on prescribed medication codes consistent with COPD. These non-diagnostic codes would likely have increased our sensitivity in identifying patients who might have had COPD, but with less certainty that a clinician had actively made that diagnosis.

The defining code list was compiled by a GP researcher (LJ), selecting clinically relevant diagnostic codes from the Read Code V2 directory, using a combined technique of Read Term "string searches" and searches through the hierarchy of individual parent Read codes. Selected diagnostic codes were derived from the Read directory "parent" codes for COPD (H3…) chronic bronchitis (H31..) and emphysema (H32..) and relevant "child" codes within the hierarchical code structure. We included three codes indicative of acute exacerbation of COPD (H3122, H3y0. and H3y1.) that the Quality Outcome Framework (QoF) incentive scheme) [5] specifically exclude (to avoid double counting of

patients and thereby duplicate payments to practices). In our experience, these are valid diagnostic codes, as they may be used when a previously undiagnosed patient presents with an acute exacerbation, sometimes requiring hospital admission. We also included four additional codes that describe rarer types of COPD (chronic emphysema due to chemical fumes and emphysema or COPD otherwise specified).

H3...	Chronic obstructive pulm.dis.
H31..	Chronic bronchitis
H310.	Simple chronic bronchitis
H3100	Chronic catarrhal bronchitis
H310z	Simple chronic bronchitis NOS
H311.	Mucopurulent chr.bronchitis
H3110	Purulent chronic bronchitis
H3111	Fetid chronic bronchitis
H311z	Mucopurulent chr.bronchit.NOS
H312.	Obstructive chronic bronchitis
H3120	Chronic asthmatic bronchitis
H3121	Emphysematous bronchitis
H3122	Acute exacerbation of COAD
H3123	Bronchiolitis obliterans
H312z	Obstructive chr.bronchitis NOS
H313.	Mixd simp+mucopur chron bronch
H31y.	Other chronic bronchitis
H31y1	Chronic tracheobronchitis
H31yz	Other chronic bronchitis NOS
H31z.	Chronic bronchitis NOS
H32..	Emphysema
H320.	Chronic bullous emphysema
H3200	Segmental bullous emphysema
H3201	Zonal bullous emphysema
H3202	Giant bullous emphysema
H3203	Bullous emphysema + collapse
H320z	Chronic bullous emphysema NOS
H321.	Panlobular emphysema
H322.	Centrilobular emphysema
H32y.	Other emphysema
H32y0	Acute vesicular emphysema
H32y1	Atrophic (senile) emphysema
H32y2	MacLeod's unilateral emphysema
H32yz	Other emphysema NOS
H32z.	Emphysema NOS
H36..	Mild chron obstr pulm disease
H37..	Mod chron obstr pulm disease
H38..	Sev chron obstr pulm disease
H39..	Very severe COPD
H3A..	End stag chron obst airway dis
H3y..	Chronic obstr.airway dis.OS
H3y0.	Chr obs pulm dis+ac l resp inf
H3y1.	Chr obs pulm dis+ac exac
H3z..	Chronic obstr.airway dis.NOS
H4640	Chronic chemical emphysema
H4641	Chemical obliter.bronchiolitis
Hyu30	[X]Other emphysema
Hyu31	[X]O spcf chron obs pulmon dis

Diagnostic Read codes used in sensitivity analyses for COPD cohort-defining codes

1. Read codes indicative of acute exacerbation of COPD:
   'H3y0.','H3y1.', 'H3122'

2. Read codes indicative of chronic bronchitis that do not *in themselves* imply the presence of airflow obstruction:
   'H31..','H310.','H311.','H313.','H31y.','H31z.', 'H3100', 'H310z', 'H3110', 'H3111', 'H311z', 'H31y1', 'H31yz'

Co-morbidities

When developing code lists defining comorbidities, we adopted a different approach and included Read codes from the "process of care" as well as the "diagnosis" chapters of the Read browser. This was to prioritise sensitivity over specificity, thereby increasing our confidence that those without codes for the comorbidity had not received this diagnosis. Code lists were created independently by three clinicians, with the final list derived by consensus, justifying inclusion of each code if "it alone suggested a very high likelihood that the patient had the comorbid disease in question". Codes for medication specific to dementia (Donepezil/Aricept) and idiopathic pulmonary fibrosis (Pirfenidone/Esbriet) were included in code lists for these comorbidities, as their use is specific to these conditions.

Heart Failure

14A6.	H/O: heart failure
14AM.	H/O:Heart failure in last year
183B.	Worsening pulmonary oedema
1O1..	Heart failure confirmed
23E1.	O/E - pulmonary oedema
33BA.	Impaired left ventricular func
388D.	NYHA classif heart fail symps
585f.	Echocardiogram shows LVSDF
585g.	Echocardiogram shows LVDDF
661M5	Heart fail slf-manag pln agree
661N5	Heart fail self-manag pln revw
662f.	NYHA classification - class I
662g.	NYHA classification - class II
662h.	NYHA classification- class III
662i.	NYHA classification - class IV
662p.	Heart failure 6 month review
662T.	Congestive heart failure monit
662W.	Heart failure annual review
679W1	Educatn deterior heart failure
679X.	Heart failure education
67D4.	Heart failure info given to pt
7936J	Imp intr biventric car pace sy
79379	Implant bivent card pacemak sy
8B29.	Cardiac failure therapy
8CeC.	Pref pl cre nxt exacerbatin HF
8CL3.	HF care plan discussed with pt
8CMK.	Has heart failure manage plan
8CMW8	Heart failure clinical pathway

8H2S.	Admit heart failure emergency
8HBE.	Heart failure follow-up
8HHb.	Referral to heart failure nurs
8HHz.	Ref to heart failur exerc prog
8Hk0.	Refd - heart fail educat group
8HTL.	Referral heart failure clinic
8HTL0	Ref rapid acess hert fail clnc
8IE0.	Ref heart fail eductn grp dcld
8IE1.	Ref heart failur exer prog dec
9hH1.	Ex heart fai qual ind: Inf dis
9N0k.	Seen in heart failure clinic
9N2p.	Seen by comm heart failur nurs
9Or0.	Heart failure review completed
G1yz1	Rheumatic left ventric.failure
G2101	Malig.hypert.heart dis.-+ CCF
G2111	Benign hypert.heart dis-+ CCF
G234.	Hyp ht&ren d+both(con)h&r fail
G58..	Heart failure
G580.	Congestive heart failure
G5800	Acute congestive heart failure
G5801	Chroncongestive heart failure
G5802	Decompensated cardiac failure
G5803	Compensated cardiac failure
G5804	Cong heart fail due valv dis
G581.	Left ventricular failure
G5810	Acute left ventricular failure
G582.	Acute heart failure
G583.	Heart failure norm eject frac
G58z.	Heart failure NOS
G5yy9	Left ventricul systol dysfunc
G5yyA	Left ventric diastolic dysfunc
G5yyC	Diastolic dysfunction
G5yyD	Lft ventr cardiac dysfunction
Q48y1	Congenital cardiac failure
R2y10	[D]Cardiorespiratory failure
SP111	Cardiac insuffic.comp.of care
SP112	Cardioresp.fail.comp.of care
ZV45M	[V]Biventric pacemaker in situ

Hypertension

6627	Good hypertension control
6628	Poor hypertension control
662b.	Moderate hypertension control
662c.	Hypertension six month review
662d.	Hypertension annual review
662O.	On treatment for hypertension
662P0	Hypertension 9 month review
8BL0.	Pt on max tol antihypert ther
G2...	Hypertensive disease
G20..	Essential hypertension
G200.	Malignant essential hypertens.
G201.	Benign essential hypertension
G202.	Systolic hypertension
G203.	Diastolic hypertension

G20z.	Essential hypertension NOS
G21..	Hypertensive heart disease
G210.	Malignant hypertens.heart dis.
G2100	Malig.hypert.heart dis.-no CCF
G2101	Malig.hypert.heart dis.-+ CCF
G210z	Malig.hypertens.heart dis. NOS
G211.	Benign hypertensive heart dis.
G2110	Benign hypert.heart dis-no CCF
G2111	Benign hypert.heart dis-+ CCF
G211z	Benign hypertens.heart dis.NOS
G21z.	Hypertensive heart disease NOS
G21z0	Hypertens.heart dis.NOS-no CCF
G21z1	Hypertens.heart dis.NOS- + CCF
G21zz	Hypertensive heart disease NOS
G22..	Hypertensive renal disease
G220.	Malignant hypertens.renal dis.
G221.	Benign hypertensive renal dis.
G222.	Hypertens renal dis+renal fail
G22z.	Hypertensive renal disease NOS
G23..	Hypertensive heart+renal dis.
G230.	Malig.hypert.heart+renal dis.
G231.	Benign hypert.heart+renal dis.
G232.	Hypert ht&ren d+(congs)ht fail
G233.	Hypertn hrt&ren dis+renal fail
G234.	Hyp ht&ren d+both(con)h&r fail
G23z.	Hypertens.heart+renal dis.NOS
G24..	Secondary hypertension
G240.	Secondary malignant hypertens.
G2400	Second.malig.renovasc.hypert.
G240z	Secondary malign.hypertens.NOS
G241.	Secondary benign hypertension
G2410	Second.benign renovasc.hypert.
G241z	Secondary benign hypertens.NOS
G244.	Hypertens 2ndry endocrin disor
G24z.	Secondary hypertension NOS
G24z0	Secondary renovasc.hypert. NOS
G24zz	Secondary hypertension NOS
G25..	Stge 1 hypertensin (NICE 2011)
G250.	Stage 1 hyp wo ev end org dmge
G251.	Stage 1 hyp wi ev end org dmge
G26..	Severe hypertensin (NICE 2011)
G27..	Hypertnsn resistnt to drg ther
G28..	Stge 2 hypertensin (NICE 2011)
G2y..	Hypertensive disease OS
G2z..	Hypertensive disease NOS
G672.	Hypertensive encephalopathy
Gyu2.	[X]Hypertensive diseases
Gyu20	[X]Oth secondary hypertension
Gyu21	[X]Hyperten

Hyperlipidaemia

44O4.	Serum lipids high
44P3.	Serum cholesterol raised
44P4.	Serum cholesterol very high

44Q3.	Serum triglycerides raised
662X.	Target cholesterol level
8BAG1	Cholesterol red progm attended
8BAG2	Cholesterol red progm declined
8BL1.	Pt on max tol lipid low ther
8CR3.	Hyperlipidaemia clin man plan
8HT1.	Referral to lipid clinic
9N0I.	Seen in lipid clinic
9N0J.	Seen in cholesterol clinic
9N4K.	DNA cholesterol clinic
9Oc0.	Attnds lipid disordr monitring
9Oc1.	Lipid disrdr monitring declind
C320.	Pure hypercholesterolaemia
C3200	Familial hypercholesterolaemia
C3201	Hyperbetalipoproteinaemia
C3202	Hyperlipidaemia
C3203	LDL hyperlipoproteinaemia
C3204	Fredrickson type IIa lipidaem
C3205	Fam defect apolipoprot B-100
C3206	Polygenic hypercholesterolemia
C320y	Pure hypercholesterolaemia OS
C320z	Pure hypercholesterolaemia NOS
C321.	Pure hyperglyceridaemia
C3210	Hypertriglyceridaemia
C322.	Mixed hyperlipidaemia
C3220	Familial comb hyperlipidaemia
C323.	Hyperchylomicronaemia
C324.	Hyperlipidaemia NOS
C325.	Lipoprotein deficiencies
C3250	High density lipoid deficiency
C3251	Hypo-alpha-lipoproteinaemia
C325z	Lipoprotein deficiency NOS
C328.	Dyslipidaemia
C329.	Hypercholesterolaemia
C32y4	Lipase deficiency
Cyu8D	[X]Other hyperlipidaemia

Osteoporosis

58EG.	Hip DXA result osteoporotic
58EM.	Lumbar DXA result osteoporotic
58EV.	Femor nec DEXA scan osteoporot
66a..	Osteoporosis monitoring
66a2.	Osteoporosis treatment started
66a4.	Osteoporosis treatment changed
66a5.	Osteoporosis - no treatment
66a6.	Osteoporosis - dietary advice
66a7.	Osteoporosis - diet assessment
66a8.	Osteoporosis - exercise advice
66a9.	Osteoporosis-falls prevention
66aA.	Osteoporosis-treatmnt response
66aB.	Osteoporosis-no treat response
9hP1.	Exc osteo qual ind: infor diss
9kj0.	Bn spr drg trt ofr osteo - ESA
9Od9.	Osteoporosis monit check done

N330.	Osteoporosis
N3300	Osteoporosis
N3301	Senile osteoporosis
N3302	Postmenopausal osteoporosis
N3303	Idiopathic osteoporosis
N3304	Dissuse osteoporosis
N3305	Drug-induced osteoporosis
N3306	Postoophorectomy osteoporosis
N3307	Postsurg malabsorp osteoporos
N3309	Osteopor
N330A	Osteoporosis in endocr disord
N330B	Vertebral osteoporosis
N330C	Osteoporosis localized spine
N330D	Osteoporos due corticosteroid
N330z	Osteoporosis NOS
N3312	Postoophorc osteopor+path frct
N3313	Osteopor of disuse + path frct
N3314	Postsur malab osteop+path frct
N3315	Drug-ind osteopor + path fract
N3316	Idiopath osteopor + path fract
N3318	Osteopor path # lumb vertebrae
N3319	Osteopor path # thor vertebrae
N331A	Osteopor path # cerv vertebrae
N331B	Postmenop osteopor+path fract
N331H	Collap cerv vert due to osteop
N331J	Collap lumb vert due to osteo
N331K	Coll thorac vert due osteopor
N331L	Collap vert due osteopor NOS
N331M	Fragility # unsp osteoporosis
N3746	Osteoporotic kyphosis
NyuB0	[X]Oth osteoporosis+patholog #
NyuB1	[X]Other osteoporosis
NyuB2	[X]Osteoporosis/oth disords CE
NyuB8	[X]Unsp osteopor + pathol frac

Cerebrovascular Disease

14AK.	H/O: Stroke in last year
1M4..	Central post-stroke pain
661M7	Stroke self-manage plan agreed
661N7	Stroke self-manage plan review
662e.	Stroke/CVA annual review
662M.	Stroke monitoring
662M1	Stroke 6 month review
662M2	Stroke initial post disch revw
662o.	Haemorrhagic stroke monitoring
7P242	Delivery rehabilitation stroke
8CRB.	TIA clinical management plan
8HHM.	Ref to stroke func improv serv
8IEC.	Ref multidis strk fnc impv dcd
9h2..	Except report: stroke qual ind
9h21.	Except stroke qual ind: Pt uns
9h22.	Exc stroke qual ind: Infor dis
E004.	Arteriosclerotic dementia
E0040	Arterioscl.dementia-uncomplic.

E0041	Arterioscl.dementia+delirium
E0042	Arterioscl.dementia+paranoia
E0043	Arterioscl.dementia+depression
E004z	Arteriosclerotic dementia NOS
Eu01.	[X]Vascular dementia
Eu010	[X]Vascular dement acute onset
Eu011	[X]Multi-infarct dementia
Eu012	[X]Subcortical vascular dement
Eu013	[X]Mix cort/subcor vasc dement
Eu01y	[X]Other vascular dementia
Eu01z	[X]Vascular dementia unspecif
F11x2	Cerebral degen cerebrovasc dis
Fyu55	[X]Oth cerebral TIA's+rel synd
Fyu57	[X]O vasc syn/brain cer vasc d
G6...	Cerebrovascular disease
G61..	Intracerebral haemorrhage
G610.	Cortical haemorrhage
G611.	Internal capsule haemorrhage
G612.	Basal nucleus haemorrhage
G613.	Cerebellar haemorrhage
G614.	Pontine haemorrhage
G615.	Bulbar haemorrhage
G616.	External capsule haemorrhage
G618.	Intracerebrl haem
G619.	Lobar cerebral haemorrhage
G61X.	Intracer haem hemisph
G61X0	Left side intracereb haem unsp
G61X1	Right side intracer haem unsp
G61z.	Intracerebral haemorrhage NOS
G63..	Precerebral arterial occlusion
G630.	Basilar artery occlusion
G631.	Carotid artery occlusion
G632.	Vertebral artery occlusion
G633.	Multip/bilat.precereb.art.occ.
G634.	Carotid artery stenosis
G63y.	Other precerebral artery occl.
G63y0	Cerebr infct/throm/precere art
G63y1	Cerebr infct/embol/precere art
G63z.	Precerebral artery occlus. NOS
G64..	Cerebral arterial occlusion
G640.	Cerebral thrombosis
G6400	Cerebr infct/throm/cerebrl art
G641.	Cerebral embolism
G6410	Cerebr infct/embol/cerebrl art
G64z.	Cerebral infarction NOS
G64z0	Brainstem infarction
G64z1	Wallenberg syndrome
G64z2	Left sided cerebral infarction
G64z3	Right sided cerebral infarct
G64z4	Infarction of basal ganglia
G65..	Transient cerebral ischaemia
G650.	Basilar artery syndrome
G651.	Vertebral artery syndrome
G6510	Vertebro-basilar artery syndrm

G653.	Carotid artery syn hemispheric
G654.	Multi&bilat precerebrl art syn
G656.	Vertebrobasilar insufficiency
G657.	Carotid terr trans ischaem att
G65y.	Other transient cerebral isch.
G65z.	Transient cerebral ischaem.NOS
G65z0	Impending CVA
G65z1	Intermittent CVA
G65zz	Transient cerebral ischaem.NOS
G66..	Stroke/CVA unspecified
G660.	Middle cerebral artery syndrm
G661.	Anterior cerebral artery syn
G662.	Posterior cerebral artery syn
G663.	Brain stem stroke syndrome
G664.	Cerebellar stroke syndrome
G665.	Pure motor lacunar syndrome
G666.	Pure sensory lacunar syndrome
G667.	Left sided CVA
G668.	Right sided CVA
G67..	Other cerebrovascular disease
G670.	Cerebral atherosclerosis
G671.	Generalised isch.CV dis.NOS
G6710	Acute cerebrovasc.insuffic.NOS
G6711	Chronic cerebral ischaemia
G671z	Generalised isch.CV dis.NOS
G677.	Oc/st cere art
G6770	Occlusn+stenos/midl cerebr art
G6771	Occlusn+stenos/anter cereb art
G6772	Occlusn+stenos/post cerebr art
G6773	Occlusn+stenos/cerebellar art
G6774	Occl/sten/mult+bilat cereb art
G679.	Small vessel cerebrovas diseas
G67B.	Revrsb cerbrl vascnstrtn syndr
G67y.	Other cerebrovascular dis OS
G67z.	Other cerebrovasc.disease NOS
G68..	Cerebrovasc.dis.-late effects
G682.	Seq/oth nontraum intrcran haem
G683.	Sequelae/cerebral infarction
G68W.	Seql/o+unspcf cerebvasc dis
G68X.	Seql/strok
G6W..	Cer inf
G6X..	Cereb in/uns oc
G6y..	Cerebrovascular disease OS
G6z..	Cerebrovascular disease NOS
G70y0	Carotid artery atherosclerosis
Gyu6.	[X]Cerebrovascular diseases
Gyu63	[X]Cereb in/uns oc
Gyu64	[X]Other cerebral infarction
Gyu65	[X]Oc+steno/o precerebral artr
Gyu66	[X]Oc+sten/o cerebral arteries
Gyu67	[X]Other spcfd cerebrovasc dis
Gyu6A	[X]Oth cerebrovasc diso/dis CE
Gyu6B	[X]Seql/o n-traum intracrn h'm
Gyu6C	[X]Seql/strok

Gyu6D	[X]Seql/o+unspcf cerebvasc dis
Gyu6G	[X]Cer inf
ZV12D	[V]Pers hist trans isch attack

Dementia

1461	H/O: dementia
3AE3.	GDS level 4 - mod cog dec
3AE4.	GDS level 5 - mod sev cog dec
3AE5.	GDS level 6 - severe cog dec
3AE6.	GDS level 7 - very sev cog dec
66h..	Dementia monitoring
6AB..	Dementia annual review
8BPa.	Antipsyc drug therapy dementia
8CMG2	Review dementia adv care plan
8CMZ.	Dementia care plan
8CSA.	Dementia advnce cre pln agreed
8Hla.	Referral dementia care advisor
8IAe0	Dementia adv care plan declnd
9hD..	Excep report: demen qual indic
9hD0.	Exc demen qual ind: Pat unsuit
9hD1.	Exc demen qual ind: Inform dis
E000.	Senile dementia-uncomplicated
E001.	Presenile dementia
E0010	Presenile dementia - uncomplic
E0011	Presenile dementia + delirium
E0012	Presenile dementia + paranoia
E0013	Presenile dementia+depression
E001z	Presenile dementia NOS
E002.	Sen.dement.-depressed/paranoid
E0020	Senile dementia + paranoia
E0021	Senile dementia + depression
E002z	Sen.dement.-depr./paranoid NOS
E003.	Senile dementia + delirium
E004.	Arteriosclerotic dementia
E0040	Arterioscl.dementia-uncomplic.
E0041	Arterioscl.dementia+delirium
E0042	Arterioscl.dementia+paranoia
E0043	Arterioscl.dementia+depression
E004z	Arteriosclerotic dementia NOS
E012.	Other alcoholic dementia
E02y1	Drug-induced dementia
E041.	Dementia in conditions EC
Eu00.	[X]Dementia in Alzheimer's
Eu000	[X]Early onset Alzheim dement
Eu001	[X]Late onset Alzheim dementia
Eu002	[X]Atypical/mixed Alzheimer's
Eu00z	[X]Alzheimer's disease unspec
Eu01.	[X]Vascular dementia
Eu010	[X]Vascular dement acute onset
Eu011	[X]Multi-infarct dementia
Eu012	[X]Subcortical vascular dement
Eu013	[X]Mix cort/subcor vasc dement
Eu01y	[X]Other vascular dementia
Eu01z	[X]Vascular dementia unspecif

Eu02.	[X]Dementia in disease EC
Eu020	[X]Dementia in Pick's disease
Eu021	[X]Dement in Creutzfeld-Jakob
Eu022	[X]Dementia in Huntington's
Eu023	[X]Dementia in Parkinson's
Eu024	[X]Dementia in HIV disease
Eu025	[X]Lewy body dementia
Eu02y	[X]Dement
Eu02z	[X] Unspecified dementia
Eu041	[X]Delirium superimp dementia
Eu107	[X]Resid psychotic due alcohol
F110.	Alzheimer's disease
F1100	Alzheimer dis wth early onset
F1101	Alzheimer's dis wth late onset
F111.	Pick's disease
F112.	Senile degeneration of brain
F116.	Lewy body disease
F11x7	Cerebral degen.-Jakob-Creutzf.
Fyu30	[X]Other Alzheimer's disease
AROR23861NEMIS	Aricept Evess Orodispersible tablets 5 mg
AROR23862NEMIS	Aricept Evess Orodispersible tablets 10 mg
ARTA30542EMIS	Aricept Tablets 10 mg
ARTA30543EMIS	Aricept Tablets 5 mg
DOOR23858NEMIS	Donepezil Hydrochloride Orodispersible Tablets (Sugar Free) 5 mg
DOOR23859NEMIS	Donepezil Hydrochloride Orodispersible Tablets (Sugar Free) 10 mg
DOTA30546EMIS	Donepezil Hydrochloride Tablets 10 mg
DOTA30547EMIS	Donepezil Hydrochloride Tablets 5 mg
dy1..	DONEPEZIL HYDROCHLORIDE
dy11.	DONEPEZIL HYDROCHLORIDE 5mg tablets
dy12.	DONEPEZIL HYDROCHLORIDE 10mg tablets
dy13.	ARICEPT 5mg tablets
dy14.	ARICEPT 10mg tablets
dy15.	ARICEPT EVESS 5mg disp tabs
dy16.	ARICEPT EVESS 10mg disp tabs
dy1y.	DONEPEZIL HYDROCHLORIDE 10mg disp tabs
dy1z.	DONEPEZIL HYDROCHLORIDE 5mg disp tabs

Peripheral Vascular Disease

14F7.	H/O: arterial lower limb ulcer
1M110	Ischaemic foot pain at rest
1M111	Ischaemic ft pain when walking
2G63.	Ischaemic toe
662U.	Periph vasc disease monitoring
7A121	Bypass bifurc anast fem a NEC
7A123	Bypass bifurc anastom iliac a
7A41.	Other bypass of iliac artery
7A411	Bypass iliac anast il/fem NEC
7A413	Bypass iliac anast fem/fem NEC
7A419	Byp comm iliac anast aort/c il
7A41A	Byp iliac art anast aort/ex il
7A41B	Byp leg art anast aorta/c fem
7A41C	Byp leg art anast aorta/d fem
7A41D	Byp iliac art anast il/il NEC
7A41E	Em bypass iliac art-unsp anast

7A41F	Ilio-fem prosth X-over graft
7A41y	Other bypass of iliac art OS
7A41z	Other bypass of iliac art NOS
7A420	Endartect patch rep iliac art
7A421	Endarterectomy iliac art NEC
7A431	Open embolectomy iliac artery
7A433	Open insert iliac artery stent
7A44.	Transluminal ops on iliac art
7A440	PC transl angioplasty iliac a
7A441	PC transl embolectomy iliac a
7A443	Insertion iliac artery stent
7A444	Per trans ins iliac art stent
7A44y	Transluminal op iliac art OS
7A44z	Transluminal op iliac art NOS
7A48.	Oth bypass femoral/poplit art
7A480	Bypass fem art pr anas/pop NEC
7A481	Bypass pop art pr anas/pop NEC
7A482	Bypass fem art vein gr/pop NEC
7A483	Bypass pop art vein gr/pop NEC
7A484	Bypass fem art pr anas/tib NEC
7A485	Bypass pop art pr anas/tib NEC
7A486	Bypass fem art vein gr/tib NEC
7A487	Bypass pop art vein gr/tib NEC
7A488	Bypass fem art pr anas/per NEC
7A489	Bypass pop art pr anas/per NEC
7A48A	Bypass fem art vein gr/per NEC
7A48B	Bypass pop art vein gr/per NEC
7A48C	Byp fem art anast fem/fem NEC
7A48D	Byp pop art anast pop/fem NEC
7A48E	Fem-fem prosth X-over graft
7A48y	Other bypass fem/poplit art OS
7A48z	Oth bypass fem/pop artery NOS
7A49.	Reconstruction fem/pop artery
7A490	Endarterect patch rep feml art
7A491	Endarterect patch rep popl art
7A492	Endarterect femoral artery NEC
7A493	Endarterect poplit artery NEC
7A494	Profundoplast patch rep fem a
7A495	Profundoplast patch rep popl a
7A496	Profundoplasty femoral art NEC
7A497	Profundoplasty poplit art NEC
7A498	Recon femoral artery vein graf
7A499	Recon popliteal art vein graft
7A49y	Reconstruction fem/pop art OS
7A49z	Reconstruction fem/pop art NOS
7A4A2	Open embolectomy femoral art
7A4A3	Open embolectomy popliteal art
7A4B.	Translum ops fem/poplit artery
7A4B0	PC TL angioplasty femoral art
7A4B1	PC TL angioplasty poplit art
7A4B2	PC TL embolectomy femoral art
7A4B3	PC TL embolectomy poplit art
7A4B8	PC TL thromb fem gr streptokin
7A4B9	Perc trans ins stent fem art

7A4By	Translum op fem/poplit art OS
7A4Bz	Translum op fem/poplit art NOS
9hS1.	Ex f pr art dis qu ind inf dis
C107.	Diab.mell.+periph.circul.dis
C1070	Diab.+periph.circ.dis-juvenile
C1071	Diab.+periph.circ.dis.-adult
C1072	Diabetic gangrene - adult
C1073	IDDM periph circulatory disord
C1074	NIDDM periph circulat disord
C107y	Oth spcf diab mel+per circ cmp
C107z	Diab.+periph.circ.disease NOS
C1086	Insulin depen diab mel+gangren
C108G	IDDM with peripheral angiopath
C1095	Non-insulin dep diab mell+gang
C109F	NIDDM with periph angiopath
C10A5	Malnut-rlt diab mel+per circ c
C10E6	Type 1 diab mell with gangrene
C10EG	Type 1 diab mell+periph angiop
C10F5	Type 2 diab mell + gangrene
C10FF	Type 2 diab mell+perip angiop
G702.	Extremity artery atheroma
G702z	Extremity artery atheroma NOS
G73..	Other peripheral vascular dis.
G731.	Thromboangiitis obliterans
G7310	Buerger's disease
G7311	Presenile gangrene
G731z	Thromboangiitis obliterans NOS
G732.	Peripheral gangrene
G7320	Gangrene of toe
G7321	Gangrene of foot
G733.	Ischaemic foot
G734.	Peripheral arterial disease
G73y.	Other spec.periph.vasc.disease
G73y0	Diabetic peripheral angiopathy
G73y1	Periph.angiopathy
G73yz	Other spec.periph.vasc.dis.NOS
G73z.	Peripheral vascular dis. NOS
G73z0	Intermittent claudication
G73zz	Peripheral vasc.disease NOS
G7424	Embolus/thrombus femoral art.
G7425	Embolus/thromb.popliteal art.
G7426	Embolus/thromb.ant.tibial art.
G7427	Embolus/thromb.dors.pedis art.
G7428	Embolus/thromb.post.tibial art
G7429	Embolus/thromb.leg artery NOS
G74y0	Embolus/thromb.com.iliac art.
G74y1	Embolus/thromb.int.iliac art.
G74y2	Embolus/thromb.ext.iliac art.
G74y3	Embolus/thromb iliac art.unsp.
G76z0	Iliac artery occlusion
G76z1	Femoral artery occlusion
G76z2	Popliteal artery occlusion
G784.	Occlusion of artery lower limb
G7840	Occlusn dorsalis pedis artery

G7841	Occlusn anterior tibial artery
G7842	Occlusn posterior tibial artry
Gyu74	[X]Oth spcf periph vasculr dis
M2710	Ischaemic ulcer diabetic foot
M2713	Arterial leg ulcer
M2714	Mixed venous+artery leg ulcer
R0542	[D]Gangrene of toe in diabetic
R0543	[D]Widespread diab foot gangr

Gastro Oesophageal Reflux

171J.	Reflux cough
1952	Regurgitates food
1953	Waterbrash
1957	Gastric reflux
760L.	Antireflux operations
760L0	Antireflux fundoplic thor appr
760L1	Antireflux op thorac appr NEC
760L2	Antireflux fundoplic abd appr
760L3	Antireflux gastropexy
760L4	Antireflux proc & gastropl HFQ
760L5	Insertion Angelchick prosthes
760L6	Oesophagogastric fundoplasty
760L7	Endo Nissen fund thoracic appr
760L8	Lap Nissen fundoplic abdo appr
760Ly	Antireflux operation OS
760Lz	Antireflux operation NOS
760M.	Revision antireflux operations
760M0	Revision fundoplication stom
760M1	Adjustment Angelchick prosthes
760M2	Removal Angelchick prosthesis
760My	Revision antireflux op OS
760Mz	Revision antireflux op NOS
J1011	Reflux oesophagitis
J1020	Peptic ulcer of oesophagus
J1025	Barrett's ulcer of oesophagus
J1034	Peptic stricture of oesophagus
J10y4	Oesoph reflux no oesophagitis
J10y5	Laryngopharyngeal reflux
J10y6	Barrett's oesophagus
R0711	[D]Waterbrash

Connective Tissue Disease

66c0.	DMARD monitoring
66HB0	Rheumatoid arthritis annul rev
7P203	Del rehab rheumatoid arthritis
9hR1.	Ex rheum arth qua ind: inf dis
9kN..	DMARD monitor - enh serv admin
9kN0.	Pt DMARD rec iss - enh ser adm
9kN1.	Date DMARD mon ap - enh ser ad
9kN2.	Pt DMARD rec upd - enh ser adm
9kN4.	Pt DMARD recor chec - en se ad
9kN5.	DMARD mn rf bk sc cr - en se a
9kN6.	DMARD monitor prmry care - ESA
9kN7.	DMARD monitor secnd care - ESA

9kN8.	DMARD therapy initiated
9NiK.	DNA hospital DMARD monitor clc
9NiL.	DNA GP DMARD monitoring clinic
9NkF.	Seen in GP DMARD monitor clinc
9NkG.	Seen in com DMARD monitor clnc
9NkH.	Seen in hosp DMARD monitr clnc
9Oe0.	Community DMARD monitor appt
9Oe3.	DNA community DMARD monitoring
9Oe4.	Co DMARD mo record retrn to pt
F3712	Polyneuropathy+rheumatoid arth
F3964	Myopathy+rheumatoid arthritis
F3966	Myopathy + scleroderma
F3967	Myopathy + Sjogren's disease
G5y8.	Rheumatoid myocarditis
G5yA.	Rheumatoid carditis
H570.	Rheumatoid lung
H572.	Lung dis.+ systemic sclerosis
H57y1	Lung dis.+ polymyositis
H57y3	Lung dis.+ Sjogren's disease
H57y4	Lung disease + SLE
H58y7	Interst lung dis conn tiss dis
K01x4	Nephrotic syndrome + SLE
K0B4.	Ren tb-in ds/sys con tis ds
K0B40	Renal tubul-interstit dis SLE
N00..	Diffuse connective tissue dis.
N000.	Systemic lupus erythematosus
N0000	Disseminated lupus erythemat.
N0003	Syst lup eryth + organ/sys inv
N0004	SLE with pericarditis
N0006	Cerebral lupus
N000z	Systemic lupus erythematos.NOS
N001.	Scleroderma
N0010	Progressive systemic sclerosis
N0011	CREST syndrome
N002.	Sicca (Sjogren's) syndrome
N003.	Dermatomyositis
N003X	Dermatopolymyositis
N004.	Polymyositis
N04..	Rheumatoid arthritis+similar
N040.	Rheumatoid arthritis
N0400	Rheumatoid arthritis-Cx spine
N0401	Oth rheumatoid arthritis-spine
N0402	Rheumatoid arthritis-shoulder
N0403	Rheumatoid arthr-sternoclav jt
N0404	Rheumatoid arthr-acromioclav j
N0405	Rheumatoid arthritis of elbow
N0406	Rheumatoid arthritis-dist RUJ
N0407	Rheumatoid arthritis of wrist
N0408	Rheumatoid arthritis-MCP joint
N0409	Rheumatoid arthritis-PIPJ-fing
N040A	Rheumatoid arthritis-DIPJ-fing
N040B	Rheumatoid arthritis of hip
N040C	Rheumatoid arthritis of SIJ
N040D	Rheumatoid arthritis of knee

N040E	Rheumatoid arthr of tib-fib jt
N040F	Rheumatoid arthritis of ankle
N040G	Rheumatoid arthr-subtalar jnt
N040H	Rheumatoid arthr-talonav joint
N040J	Rheumatoid arthr-oth tarsal jt
N040K	Rheumatoid arthr-1st MTP joint
N040L	Rheumatoid arthr-lesser MTP jt
N040M	Rheumatoid arthr-IP joint-toe
N040N	Rheumatoid vasculitis
N040P	Seronegative rheumat arthritis
N040Q	Rheumatoid bursitis
N040S	Rheumat arthr - multiple joint
N040T	Flare of rheumatoid arthritis
N041.	Felty's syndrome
N042.	Other rh.arthr.+visc/syst.dis.
N0421	Rheumatoid lung disease
N042z	Rh.arthr.+visc/syst.dis.NOS
N047.	Seropositive erosive RA
N04X.	Seroposit rheum arthr
N04y0	Rheumatoid lung
N2334	Antisynthetase syndrome
N33z5	Relapsing polychondritis
Nyu10	[X]Rheum arthrit+inv/o org/sys
Nyu11	[X]O sero+ve rheumat arthritis
Nyu12	[X]Oth spcf rheumatd arthritis
Nyu1G	[X]Seroposit rheum arthr
Nyu4.	[X]Systmc connctv tis disordrs
Nyu43	[X]Oth forms/sys lup erythemat
Nyu44	[X]Other dermatomyositis
Nyu45	[X]Oth forms/systemc sclerosis
Nyu46	[X]Other overlap syndromes
Nyu47	[X]Oth syst dis/connctv tissue
Nyu48	[X]Dermat(poly)myosit/neo d CE
Nyu4C	[X]Sys diso/connctv t/o dis CE
Nyu4E	[X]Dermatopolymyositis
Nyu4F	[X]Mixed connect tiss disease

Anxiety/Depression

1465	H/O: depression
1466	H/O: anxiety state
173f.	Anxiety about breathlessness
1B13.	Anxiousness
1B17.	Depressed
1B1U.	Symptoms of depression
1B1V.	C/O - panic attack
1BT..	Depressed mood
1JJ..	Suspected depression
212S.	Depression resolved
2257	O/E - depressed
38Dp.	HAMD-Hamil rating scal depress
62T1.	Puerperal depression
66590	Antidepress drug treat started
6G00.	Postnatal depression counsel
8BK0.	Depression management program

8CAa.	Pt given adv manag depression
8G94.	Anxiety management training
8HHp.	Ref guid self-help for anxiety
8HHq.	Ref guid self-help for depress
9H90.	Depression annual review
9H91.	Depression medication review
9H92.	Depression interim review
9HA0.	On depression register
9hC1.	Exc depressi qual ind: Inf dis
9k4..	Depression - enhanc ser admin
9k40.	Depression - enh serv complete
9kQ..	On fl ds lg trt dep - en se ad
E0013	Presenile dementia+depression
E002.	Sen.dement.-depressed/paranoid
E0021	Senile dementia + depression
E0043	Arterioscl.dementia+depression
E02y3	Drug-induced depressive state
E11..	Affective psychoses
E112.	Single major depressive episod
E1120	Single major depression-unspec
E1121	Single major depression-mild
E1122	Single major depress.-moderate
E1123	Single major depression-severe
E1124	Single maj.depress.severe+psyc
E1125	Single maj.depres.-part remiss
E1126	Single maj.depres.-full remiss
E112z	Single major depression NOS
E113.	Recurrent major depressive epi
E1130	Recurr.major depression-unspec
E1131	Recurr.major depression-mild
E1132	Recurr.major depress.-moderate
E1133	Recurr.major depression-severe
E1134	Recurr.maj.depres.-severe+psyc
E1135	Recurr.maj.depres.-part remiss
E1136	Recurr.maj.depres.-full remiss
E1137	Recurrent depression
E113z	Recurr. major depression NOS
E114.	Bipolar affective - now manic
E1140	Manic bipolar affective-unspec
E1141	Manic bipolar affective-mild
E1142	Manic bipolar affect.-moderate
E1143	Manic bipolar affect.-severe
E1144	Manic bipol.affect.severe+psyc
E1145	Manic bipol.affect.part remiss
E1146	Manic bipol.affect.full remiss
E114z	Manic bipolar affective NOS
E115.	Bipolar affective - now depres
E1150	Depressed bipolar affect.-unsp
E1151	Depress.bipolar affect.-mild
E1152	Depr.bipolar affect.-moderate
E1153	Depr.bipolar affect.-severe
E1154	Depr.bipol.affect.-severe+psyc
E1155	Depr.bipol.affect.-part remiss
E1156	Depr.bipol.affect.-full remiss

E115z	Depressed bipolar affect. NOS
E116.	Mixed bipolar affective disord
E1160	Mixed bipolar affective-unspec
E1161	Mixed bipolar affective-mild
E1162	Mixed bipolar affect.-moderate
E1163	Mixed bipolar affect.-severe
E1164	Mixed bipol.affect.severe+psyc
E1165	Mixed bipol.affect.part remiss
E1166	Mixed bipol.affect.full remiss
E116z	Mixed bipolar affective NOS
E117.	Unspec bipolar affect disord
E1170	Unspecified bipolar affective
E1171	Unsp.bipolar affective-mild
E1172	Unsp.bipolar affect.-moderate
E1173	Unsp.bipolar affect.-severe
E1174	Unsp.bipol.affect.-severe+psyc
E1175	Unsp.bipol.affect.-part remiss
E1176	Unsp.bipol.affect.-full remiss
E117z	Unspecif.bipolar affective NOS
E118.	Seasonal affective disorder
E11y.	Other manic-depressive psychos
E11y0	Unspec manic-depressive psycho
E11y2	Atypical depressive disorder
E11y3	Other mixed manic-depres psych
E11z2	Masked depression
E130.	Reactive depressive psychosis
E135.	Agitated depression
E200.	Anxiety states
E2000	Anxiety state unspecified
E2001	Panic disorder
E2002	Generalised anxiety disorder
E2003	Anxiety with depression
E2004	Chronic anxiety
E2005	Recurrent anxiety
E200z	Anxiety state NOS
E202.	Phobic disorders
E204.	Neurotic (reactive) depression
E2112	Depressive personality disord
E290.	Brief depressive reaction
E290z	Brief depressive reaction NOS
E291.	Prolonged depressive reaction
E2B..	Depressive disorder NEC
E2B1.	Chronic depression
E2D0.	Anxiety/fear child/adoles.dis.
E2D00	Child/adolesc.overanxious.dis.
E2D01	Child/adolesc.fearfulness dis.
E2D0z	Anxiety/fear child/adolesc.NOS
Eu054	[X]Organic anxiety disorder
Eu204	[X]Post-schizophrenic depressn
Eu251	[X]Schzaffctve dis depres type
Eu31.	[X]Bipolar affective disorder
Eu310	[X]Bipol affec current hypoman
Eu311	[X]Bipol aff
Eu312	[X]Bipol affect manic+psychos

Eu313	[X]Bipol aff mild/mod depress
Eu314	[X]Bipol AD
Eu315	[X]Bipol aff sev depress/psych
Eu316	[X]Bipol affective dis
Eu317	[X]Bipol affect dis remission
Eu318	[X]Bipol affect disord type I
Eu319	[X]Bipol affect disord type II
Eu31y	[X]Oth bipolar affectve disord
Eu32.	[X]Depressive episode
Eu320	[X]Mild depressive episode
Eu321	[X]Moderate depressve episode
Eu322	[X]Severe depressiv no psychot
Eu323	[X]Severe depressive + psychot
Eu324	[X]Mild depression
Eu325	[X]Major depression
Eu326	[X]Major depression
Eu327	[X]Maj dep
Eu328	[X]Maj dep
Eu329	[X]Sin ma dep ep sev ps rem
Eu32A	[X]Rec ma dep ep sev ps rem
Eu32B	[X]Antenatal depression
Eu32y	[X]Other depressive episodes
Eu32z	[X]Depressive episode
Eu33.	[X]Recurrent depressive disord
Eu330	[X]Recurr depress current mild
Eu331	[X]Recurr depress current mod
Eu332	[X]Recurr dep now sever no psy
Eu333	[X]Recurr dep now sever+psych
Eu334	[X]Recurr depress in remission
Eu33y	[X]Oth recurr depressve disord
Eu33z	[X]Recurrent depress dis unsp
Eu341	[X]Dysthymia
Eu3y1	[X]Oth recurr mood affect dis
Eu40.	[X]Phobic anxiety disorders
Eu40y	[X]Other phobic anxiety disord
Eu40z	[X]Phobic anxiety disordr unsp
Eu41.	[X]Other anxiety disorders
Eu410	[X]Panic episodic paroxysm anx
Eu411	[X]Generalized anxiety disord
Eu412	[X]Mixed anxiety/depressve dis
Eu413	[X]Other mixed anxiety disord
Eu41y	[X]Other specif anxiety disord
Eu41z	[X]Anxiety disord unspecified
Eu53.	[X]Puerperal mental disord NEC
Eu530	[X]Mild puerperal ment dis NEC
Eu531	[X]Sever puerper ment dis NEC
Eu606	[X]Anxious
Eu920	[X]Depressive conduct disorder
Eu931	[X]Phobic anxiet dis childhood
Eu932	[X]Social anx dis childhood
ZV111	[V]PH - Affective disorder

Lung Cancer

B22..	Malig neop trachea/bronch/lung

B220.	Malig neop trachea
B2201	Malig neop mucosa of trachea
B220z	Malig neop trachea NOS
B221.	Malig neop main bronchus
B2210	Malig neop carina of bronchus
B2211	Malig neop hilus of lung
B221z	Malig neop main bronchus NOS
B222.	Malig neop upp lobe bronc/lung
B2220	Malig neop upper lobe bronchus
B2221	Malig neop upper lobe of lung
B222z	Malig neop upp bronc/lung NOS
B223.	Malig neop mid lobe bronc/lung
B2230	Malig neop mid lobe bronchus
B2231	Malig neop middle lobe of lung
B223z	Malig neop mid lobe bronc/lung
B224.	Malig neop low lobe bronc/lung
B2240	Malig neop lower bronchus
B2241	Malig neop lower lobe of lung
B224z	Malig neop low lobe bronc/lung
B225.	Mal neop
B22y.	Malig neop oth site bronc/lung
B22z.	Malig neop bronchus/lung NOS
BB5S2	[M]Bronchiolo-alveolar adenoca
BB5S4	[M]Alveolar adenocarcinoma
Byu20	[X]Mal neop/bronchus

Chronic Kidney Disease

1Z1..	Chronic renal impairment
1Z10.	Chronic kidney disease stage 1
1Z11.	Chronic kidney disease stage 2
1Z12.	Chronic kidney disease stage 3
1Z13.	Chronic kidney disease stage 4
1Z14.	Chronic kidney disease stage 5
1Z15.	Chronic kidney diseas stage 3A
1Z16.	Chronic kidney diseas stage 3B
1Z17.	CKD stage 1 with proteinuria
1Z18.	CKD stage 1 wthout proteinuria
1Z19.	CKD stage 2 with proteinuria
1Z1A.	CKD stage 2 wthout proteinuria
1Z1B.	CKD stage 3 with proteinuria
1Z1C.	CKD stage 3 wthout proteinuria
1Z1D.	CKD stage 3A with proteinuria
1Z1E.	CKD stge 3A without proteinuria
1Z1F.	CKD stage 3B with proteinuria
1Z1G.	CKD stge 3B wthout proteinuria
1Z1H.	CKD stage 4 with proteinuria
1Z1J.	CKD stage 4 wthout proteinuria
1Z1K.	CKD stage 5 with proteinuria
1Z1L.	CKD stage 5 wthout proteinuria
661M2	CKD self-managemnt plan agreed
661N2	CKD self-manage plan review
66i..	CKD monitoring
6AA..	Chronic kid dis annual review
7A606	Creation graft fist dialysis

7A619	Ligat arterioven dialysis fist
7A61A	Ligat arterioven dialysis grft
7B00.	Transplantation of kidney
7B000	Autotransplant of kidney
7B001	Live donor kidney transplant
7B002	Cadaver donor kidney transplnt
7B003	Allotra kidney cad
7B004	Allot kid cad
7B005	Allotranspl kidney cadaver NEC
7B006	Xenograft renal transplant
7B00y	Transplantation of kidney OS
7B00z	Transplantation of kidney NOS
7B012	Bilateral nephrectomy
7B063	Exploratn of renal transplant
7B0F1	Pre-trans kid work-up
7B0F3	Post-tran kidney exam
7L1A.	Compensation for renal failure
7L1A0	Renal dialysis
7L1A1	Peritoneal dialysis
7L1A2	Haemodialysis NEC
7L1A4	Automated peritoneal dialysis
7L1A5	Cont ambulat periton dialysis
7L1A6	Peritoneal dialysis NEC
7L1Ay	Compensation for renal fail OS
7L1Az	Compensation renal failure NOS
7L1B.	Place ambu app comp renal fail
7L1B0	Insert ambul perit dial cathet
7L1B1	Remove ambul perit dial cathet
7L1B2	Flush peritoneal dialysis cath
7L1By	Pl amb app comp ren fail OS
7L1Bz	Pl amb app comp ren fail NOS
7L1C.	Place oth app comp renal fail
7L1Cy	Place app comp ren fail OS
7L1Cz	Place app comp ren fail NOS
8L50.	Renal transplant planned
9hE0.	Ex ch kid dis qu ind: Pat uns
9hE1.	Ex ch kid dis qua ind: Inf dis
C104.	Diab.mell. with nephropathy
C1040	Diab.mell.+nephrop - juvenile
C1041	Diab.mell.+nephropathy - adult
C104y	Oth specfd diab mel+renal comp
C104z	Diab.mell.+nephropathy NOS
C1080	Insuln-dep diab mel+renal comp
C1090	Non-ins-dp diab mel+renal comp
C109C	NIDDM with nephropathy
C10A2	Malnut-rlt diab mel+renal comp
C10E0	Type 1 d m with renal comps
C10ED	Type 1 diab mell + nephropathy
C10F0	Type 2 diab mell + renal compl
C10FC	Type 2 diab mell + nephropathy
D215.	Anaemia second renal failure
D2150	Anaemia secondary to CRF
G22..	Hypertensive renal disease
G220.	Malignant hypertens.renal dis.

G221.	Benign hypertensive renal dis.
G222.	Hypertens renal dis+renal fail
G22z.	Hypertensive renal disease NOS
G23..	Hypertensive heart+renal dis.
G230.	Malig.hypert.heart+renal dis.
G231.	Benign hypert.heart+renal dis.
G232.	Hypert ht&ren d+(congs)ht fail
G233.	Hypertn hrt&ren dis+renal fail
G234.	Hyp ht&ren d+both(con)h&r fail
G23z.	Hypertens.heart+renal dis.NOS
G72D.	Aneurysm dialysis AV fistula
G72D0	Anrym sprfls atry dlys AV fstl
G72D1	Anurysm nedl st dlys AV fistul
G72D2	Anurysm anst st dlys AV fistul
Gy10.	Stenosis dialysis AV graft
Gy11.	Stenosis dialysis AV shunt
Gy110	Stenosis art side dia AV shunt
Gy111	Stenosis ven side dial AV sunt
Gy20.	Thromb dialysis AV graft
Gy21.	Thromb dialysis AV fistula
Gy22.	Thromb dialysis AV shunt
Gy3..	Occlusion dialysis vasc access
Gy30.	Occl dialysis AV graft
Gy31.	Occl dialysis AV fistula
Gy32.	Occl dialysis AV shunt
Gy40.	Infect dialysis AV graft
Gy41.	Infect dialysis AV fistula
Gy42.	Infect dialysis AV shunt
Gy50.	Haemorrhage dialysis AV graft
Gy51.	Haemorrhge dialysis AV fistula
Gy52.	Haemorrhage dialysis AV shunt
Gy60.	Rupture dialysis AV graft
Gy61.	Rupture dialysis AV fistula
Gy62.	Rupture dialysis AV shunt
K05..	Chronic renal failure
K050.	End stage renal failure
K051.	Chronic kidney disease stage 1
K052.	Chronic kidney disease stage 2
K053.	Chronic kidney disease stage 3
K054.	Chronic kidney disease stage 4
K055.	Chronic kidney disease stage 5
K080.	Renal osteodystrophy
K080z	Renal osteodystrophy NOS
K08yA	Proteinuric diabetic nephrop
K0D..	End-stage renal disease
K0E..	Acute-on-chronic renal failure
K13C.	Chr drug-induced renal disease
Kyu03	[X]Glomerulr disordrs/diab mel
Kyu21	[X]Other chronic renal failure
SP06B	Contin amb per dial ass perit
SP07G	Sten arterioven dialysis fist
SP083	Kidney transplant fail+rejectn
SP08C	Accel reject renal transplant
SP08E	Acut reject renal trans grad I

SP08F	Acu reject renal trans grad II
SP08G	Acu reject rena trans grad III
SP08H	Acute reject renal transplant
SP08J	Chr rejection
SP08K	Chr rejec
SP08L	Chr rejec
SP08M	Chr rejec
SP08N	Unexplaind renal trans dysfunc
SP08R	Renal transplant rejection
SP08T	Urological complicatn renal Tx
SP08W	Vasc complictn renal transplnt
SP0E.	Disorders of PD
SP0E0	Bloodstained PD effluent
SP0E1	Thrombus in PD catheter
TB001	Kidney transplant+complication
ZV420	[V]Kidney transplanted
ZV451	[V]Renal dialysis status
ZV56.	[V]Aftercare+intermit.dialysis
ZV560	[V]Aftercare+extracorp.dialys.
ZV561	[V]Preparatory care/dialysis
ZV56y	[V]OS aftercare+intermit.dialy
ZV56z	[V]Uns.aftercare+intermit.dial

Obstructive Sleep Apnoea

Fy03.	Sleep apnoea
H5B0.	Obstructive sleep apnoea

Rhinosinusitis

2D33.	O/E - nasal polyp present
74029	Excision polyp nasal septum
74060	Nasal polypectomy
74116	Removal of antrochoanal polyp
7416D	FESS/Post op remov polyps (LA)
7416F	FESS - polypectomy nasal sinus
H11..	Nasal polyps
H110.	Polyp of nasal cavity
H1100	Choanal polyp
H110z	Polyp of nasal cavity NOS
H111.	Polypoid sinus degeneration
H1110	Woakes' ethmoiditis
H111z	Polypoid sinus degenerat.NOS
H11y.	Other polyp of sinus
H11y0	Polyp of frontal sinus
H11y1	Polyp of ethmoidal sinus
H11y2	Polyp of maxillary sinus
H11y3	Polyp of sphenoidal sinus
H11yz	Other polyp of sinus NOS
H11z.	Nasal polyp NOS
H120.	Chronic rhinitis
H1200	Chronic simple rhinitis
H1201	Chronic catarrhal rhinitis
H1202	Chronic hypertrophic rhinitis
H1203	Chronic atrophic rhinitis
H1204	Chronic infective rhinitis

H1205	Chronic ulcerative rhinitis
H1206	Chronic membranous rhinitis
H1207	Chronic fibrinous rhinitis
H120z	Chronic rhinitis NOS
H13..	Chronic sinusitis
H130.	Chronic maxillary sinusitis
H131.	Chronic frontal sinusitis
H132.	Chronic ethmoidal sinusitis
H133.	Chronic sphenoidal sinusitis
H135.	Recurrent sinusitis
H13y.	Other chronic sinusitis
H13y0	Chronic pansinusitis
H13yz	Other chronic sinusitis NOS
H13z.	Chronic sinusitis NOS
H17..	Allergic rhinitis
H170.	Allergic rhinitis - pollens
H171.	Allerg.rhinit.-other allergens
H172.	Allergic rhinitis-unsp allerg
H17z.	Allergic rhinitis NOS
Hyu21	[X]Other allergic rhinitis
Hyu22	[X]Other chronic sinusitis
Hyu23	[X]Other polyp of sinus

Bronchiectasis

A115.	Tuberculous bronchiectasis
H34..	Bronchiectasis
H340.	Recurrent bronchiectasis
H341.	Post-infective bronchiectasis
H34z.	Bronchiectasis NOS
P861.	Congenital bronchiectasis

Diabetes Mellitus

2BBF.	Retina abnormal - diabet relat
2BBk.	O/E- R st treat prol diab ret
2BBl.	O/E- L st treat prol diab ret
2BBL.	O/E - diabet maculop both eyes
2BBo.	O/E - sight threat diab retin
2BBP.	O/E - right eye back diab ret
2BBQ.	O/E - left eye back diab ret
2BBr.	Impair vision due diab retinop
2BBR.	O/E - R eye preprolif diab ret
2BBS.	O/E - L eye preprolif diab ret
2BBT.	O/E - R eye prolif diab ret
2BBV.	O/E - L eye prolif diab ret
2BBW.	O/E - R eye diab maculopathy
2BBX.	O/E - L eye diab maculopathy
2G510	Foot abnormal-diabetes related
2G5A.	O/E-Right diabet foot at risk
2G5B.	O/E-Left diabet foot at risk
2G5C.	Foot abnormal-diabetes related
2G5d.	O/E - L diab foot at incre rsk
2G5e.	O/E - R diab foot at incre rsk
2G5E.	O/E - R diab foot at low risk
2G5F.	O/E - R diab foot at mod risk

2G5G.	O/E - R diab foot at high risk
2G5H.	O/E - R diab foot - ulcerated
2G5I.	O/E - L diab foot at low risk
2G5J.	O/E - L diab foot at mod risk
2G5K.	O/E - L diab foot at high risk
2G5L.	O/E - L diab foot - ulcerated
2G5V.	O/E - R chron diab foot ulcer
2G5W.	O/E - L chron diab foot ulcer
661M4	Diabet self-manage plan agreed
661N4	Diabetes self-manage pln revw
66A2.	Follow-up diabetic assessment
66A3.	Diabetic on diet only
66A4.	Diabetic on oral treatment
66A5.	Diabetic on insulin
66AH.	Diabetic treatment changed
66AH0	Conversion to insulin
66AH1	Convrsn to insulin in 2ry care
66AH2	Convrsn to insul diab spec nrs
66AH3	Conversn non-insulin injet med
66Ai.	Diabetic 6 month review
66AI.	Diabetic - good control
66AJ.	Diabetic - poor control
66AJ1	Brittle diabetes
66AJz	Diabetic - poor control NOS
66AK.	Diabetic - cooperative patient
66AL.	Diabetic-uncooperative patient
66Am.	Insulin dose changed
66An.	Diabetes type 1 review
66AN.	Date diabetic treatment start
66Ao.	Diabetes type 2 review
66Ap.	Insulin treatment initiated
66AP.	Diabetes: practice programme
66AQ.	Diabetes: shared care program.
66AQ1	Decl conse diab year care prog
66AR.	Diabetes management plan given
66As.	Diab on subcutaneous treatment
66AS.	Diabetic annual review
66AS0	Diabetes Yr of Cre annual revw
66At0	Type I diabetic dietary review
66At1	Type II diabetic dietary revie
66Au.	Diab erectile dysfunction rev
66AU.	Diabetes care by hospital only
66Av.	Diabetic ass erect dysfunction
66AV.	Diabetic on insulin+oral treat
66o0.	Incretin mimetic treatmnt strt
679c.	Insulin administratn education
679L0	Educa self management diabetes
679L2	Education diabetes and driving
679R.	Pt offered diab struct ed prog
67D8.	Provisn diab clin summary
7276	Pan retinal photocoag diabetes
7L100	Contin subcut infusion insulin
7L198	Subcutaneous injection insulin
8A12.	Diabetic crisis monitoring

8A13.	Diabetic stabilisation
8B3l.	Diabetes medication review
8BAi.	Insulin passport completed
8BAj.	Inf dis not carry insulin pass
8BAm.	Insulin passport checked
8BL2.	Pt on max tol ther for diabet
8CE02	Insulin passport given
8CMW7	Diabetes clinical pathway
8CP2.	Transition DM care opt discuss
8CR2.	Diabetes clin management plan
8CS0.	Diabetes care plan agreed
8H2J.	Admit diabetic emergency
8H3O.	Non-urgent diabetic admission
8HBG.	Diab retinopathy 12 mth review
8HKE.	Diabetology D.V. requested
8HLE.	Diabetology D.V. done
8HME.	Listed for Diabetology admissn
8I3k.	Insulin therapy declined
9h42.	Excep diabet qual ind: Inf dis
9kL..	Insulin init - enh serv admin
9m07.	Exc diab ret scr undr ophthalm
9m08.	Exclu diab ret screen as blind
9N1Q.	Seen in diabetic clinic
9NN9.	Under care of diab spec nurse
9OL1.	Attends diabetes monitoring
9OL2.	Refuses diabetes monitoring
9OLA.	Diabetes monitor. check done
9OLD.	Diabet pt unsuit dig ret photo
C10..	Diabetes mellitus
C100.	Diab.mell. - no complication
C1000	Diab.mell.no comp. - juvenile
C1001	Diab.mell.no comp. - adult
C100z	Diab.mell.no comp. - onset NOS
C101.	Diab.mell.with ketoacidosis
C1010	Diab.mell.+ketoacid - juvenile
C1011	Diab.mell.+ketoacid - adult
C101y	Oth specfd diab mel+ketoacidos
C101z	Diab.mell.+ketoacid -onset NOS
C102.	Diab.mell. + hyperosmolar coma
C1020	Diab.mell+hyperosm.coma-juveni
C1021	Diab.mell.+hyperosm.coma-adult
C102z	Diabetes+hyperosmolar coma NOS
C103.	Diab.mell. + ketoacidotic coma
C1030	Diab.mell.+ketoac coma-juvenil
C1031	Diab.mell.+ketoac coma - adult
C103y	Oth specif diab mell with coma
C103z	Diab.mell.+ketoac coma NOS
C104.	Diab.mell. with nephropathy
C1040	Diab.mell.+nephrop - juvenile
C1041	Diab.mell.+nephropathy - adult
C104y	Oth specfd diab mel+renal comp
C104z	Diab.mell.+nephropathy NOS
C105.	Diab.mell.+ eye manifestation
C1050	Diab.mell.+eye manif -juvenile

C1051	Diab.mell.+eye manif - adult
C105y	Oth specfd diab mel+ophth comp
C105z	Diab.mell.+eye manif NOS
C106.	Diab.mell. with neuropathy
C1060	Diab.mell.+neuropathy-juvenile
C1061	Diab.mell.+neuropathy - adult
C106y	Oth specf diab mel+neuro comps
C106z	Diab.mell.+neuropathy NOS
C107.	Diab.mell.+periph.circul.dis
C1070	Diab.+periph.circ.dis-juvenile
C1071	Diab.+periph.circ.dis.-adult
C1072	Diabetic gangrene - adult
C1073	IDDM periph circulatory disord
C1074	NIDDM periph circulat disord
C107y	Oth spcf diab mel+per circ cmp
C107z	Diab.+periph.circ.disease NOS
C108.	Insulin depnd diabetes melitus
C1080	Insuln-dep diab mel+renal comp
C1081	Insul-dep diab mel+ophth comps
C1082	Insul-dep diab mel+neuro comps
C1083	Insul dep diab mel+multi comps
C1084	Unstab insul depend diab mell
C1085	Insul depen diab mel+ulcer
C1086	Insulin depen diab mel+gangren
C1087	Insul-depend diab mell+retinop
C1088	Insul dep diab mell-poor contr
C1089	Insulin dep diabet adult onset
C108A	Insulin-dependent dm no comp
C108B	IDDM with mononeuropathy
C108C	IDDM with polyneuropathy
C108D	IDDM with nephropathy
C108E	IDDM with hypoglycaemic coma
C108F	IDDM with diabetic cataract
C108G	IDDM with peripheral angiopath
C108H	IDDM with arthropathy
C108J	IDDM with neuropath arthropath
C108y	Oth specf diab mel+multip comp
C108z	Unspecifd diab mel+multip comp
C109.	Non-insulin depd diabetes mell
C1090	Non-ins-dp diab mel+renal comp
C1091	Non-ins-dp diab mel+ophth comp
C1092	Non-ins-dp diab mel+neuro comp
C1093	Non-ins-dp diab mel+multi comp
C1094	Non-insul depen diab mel+ulcer
C1095	Non-insulin dep diab mell+gang
C1096	Non-insul dep diab mel+retinop
C1097	Non-insul dep diab-poor contr
C1099	Non-insul-dep diab mel no comp
C109A	NIDDM with mononeuropathy
C109B	NIDDM with polyneuropathy
C109C	NIDDM with nephropathy
C109D	NIDDM with hypoglycaemic coma
C109E	NIDDM with diabetic cataract
C109F	NIDDM with periph angiopath

C109G	NIDDM with arthropathy
C109H	NIDDM with neuropath arthrop
C109J	Insul treated Type 2 diab mell
C109K	Hyperos non-ket stat typ 2 d m
C10A.	Malnutritn-relat diab mellitus
C10A0	Malnutrtn-reltd diab mell+coma
C10A1	Malnut-rlat diab mell+ketoacid
C10A2	Malnut-rlt diab mel+renal comp
C10A3	Malnut-rlt diab mel+ophth comp
C10A4	Malnut-rlt diab mel+neuro comp
C10A5	Malnut-rlt diab mel+per circ c
C10A6	Malnut-rlt diab mel+multi comp
C10A7	Malnut-rlt diab mel w'out comp
C10AW	Maln-rel diab m + unsp comp
C10AX	Maln-rel diab m+ot sp comps
C10B.	Diabet mel induced by steroids
C10B0	Sterod ind diab mel w/out comp
C10C.	Diab mell aut dom
C10D.	Diab mell aut dom type 2
C10E.	Type 1 diabetes mellitus
C10E0	Type 1 d m with renal comps
C10E1	Type 1 diab mell + ophth comps
C10E2	Type 1 diab mell + neuro comps
C10E3	Type 1 diab mell + mult comps
C10E4	Unstab type 1 diabet mellitus
C10E5	Type 1 diab mell with ulcer
C10E6	Type 1 diab mell with gangrene
C10E7	Type 1 diab mell + retinopathy
C10E8	Type 1 diab mell poor control
C10E9	Type 1 diab mell matur onset
C10EA	Type 1 diab mell without comp
C10EB	Type 1 diab mell + mononeurop
C10EC	Type 1 diab mell + polyneurop
C10ED	Type 1 diab mell + nephropathy
C10EE	Type 1 diab mell + hypo coma
C10EF	Type 1 diab mell + diab catar
C10EG	Type 1 diab mell+periph angiop
C10EH	Type 1 diab mell + arthropathy
C10EJ	Type 1 diab mell+neuro arthrop
C10EK	Type 1 d m + persist proteinur
C10EL	Type 1 d m + persist microalb
C10EM	Type 1 d m with ketoacidosis
C10EN	Type 1 d m+ketoacidotic coma
C10EP	Type 1 d m + exudat maculopath
C10EQ	Type 1 dm with gastroparesis
C10ER	Latent autoimm diab mell adult
C10F.	Type 2 diabetes mellitus
C10F0	Type 2 diab mell + renal compl
C10F1	Type 2 diab mell+ophthal comp
C10F2	Type 2 diab mell + neurol comp
C10F3	Type 2 diab mell + multip comp
C10F4	Type 2 diab mell with ulcer
C10F5	Type 2 diab mell + gangrene
C10F6	Type 2 diab mell + retinopathy

C10F7	Type 2 diab mell+poor control
C10F9	Type 2 diab mell without comp
C10FA	Type 2 diab mell mononeurop
C10FB	Type 2 diab mell + polyneurop
C10FC	Type 2 diab mell + nephropathy
C10FD	Type 2 diab mell+hypogly coma
C10FE	Type 2 diab mell+diab catarct
C10FF	Type 2 diab mell+perip angiop
C10FG	Type 2 diab mell + arthropathy
C10FH	Type 2 diab mell neurop+arthr
C10FJ	Insul treated Type 2 diab mell
C10FK	Hyperos non-ket stat typ 2 d m
C10FL	Type 2 d m + persist proteinur
C10FM	Type 2 d m + persist microalb
C10FN	Type 2 d m with ketoacidosis
C10FP	Type 2 d m+ketoacidotic coma
C10FQ	Type 2 d m + exudat maculopath
C10FR	Type 2 dm with gastroparesis
C10FS	Matern inherited diabetes mell
C10G.	Secondary pancreatic dm
C10G0	Second pancr dm without comp
C10H.	DM induced by non-steroid drug
C10H0	DM ind non-ster dru withou com
C10M.	Lipoatrophic diabetes mellitus
C10M0	Lipoatrophic dm without comp
C10N.	Secondary diabetes mellitus
C10N0	Secondary d m without comp
C10N1	Cyst fibro relat diab mellitus
C10y.	Diab.mell.+other manifestation
C10y0	Diab.mell.+oth manif.-juvenile
C10y1	Diab.mell.+other manif. -adult
C10yy	Oth spec diab mel+oth spec cmp
C10yz	Diab.mell.+other manifest NOS
C10z.	Diab.mell. + unspec comp
C10z0	Diab.mell.+comp NOS - juvenile
C10z1	Diab.mell.+comp NOS - adult
C10zy	Oth specf diab mel+unspec comp
C10zz	Diab.mell. + unspec comp NOS
C11y0	Steroid induced diabetes
Cyu2.	[X]Diabetes mellitus
Cyu20	[X]Oth specf diabetes mellitus
Cyu21	[X]Maln-rel diab m+ot sp comps
Cyu22	[X]Maln-rel diab m + unsp comp
Cyu23	[X]Unspec diab mel + ren compl
F1711	Autonomic neuropathy-diabetes
F3450	Diabet mononeuritis multiplex
F35z0	Diabetic mononeuritis NOS
F372.	Polyneuropathy in diabetes
F3720	Acute painful diab neuropathy
F3721	Chron painful diab neuropathy
F3722	Asymptomatic diab neuropathy
F3813	Myasthenic syndrome+diabetes
F3y0.	Diabetic mononeuropathy
F420.	Diabetic retinopathy

F4200	Background diabetic retinopath
F4201	Proliferative diabetic retinop
F4202	Preproliferative diabetic ret
F4203	Advanced diabetic maculopathy
F4204	Diabetic maculopathy
F4205	Advanced diabetic retinal dis
F4206	Non prolif diab retinop
F4207	High risk prolif diab retinop
F4208	High risk non prolif dia retin
F420z	Diabetic retinopathy NOS
F4213	Hypertensive retinopathy
F4407	Diabetic iritis
G73y0	Diabetic peripheral angiopathy
K01x1	Nephrotic syndrome+diabetes M.
K08yA	Proteinuric diabetic nephrop
Kyu03	[X]Glomerulr disordrs/diab mel
L1805	Pre-ex diab mell/insuln-depend
L1806	Pre-ex diab mel non insuln-dep
L1807	Pre-ex malnutrtn-rlat diab mel
L180X	Pre-existing diab mel
M0372	Cellulitis in diabetic foot
M21yC	Insulin lipohypertrophy
M2710	Ischaemic ulcer diabetic foot
M2711	Neuropathic diab ulcer - foot
M2712	Mixed diabetic ulcer - foot
N0300	Diabetic cheiroarthropathy
N0301	Diabetic Charcot arthropathy
PKyP.	Diab ins
R0542	[D]Gangrene of toe in diabetic
R0543	[D]Widespread diab foot gangr
ZV6DA	[V]Admitted commence insulin
ZV6DB	[V]Admitted conversion insulin

Asthma

173A.	Exercise induced asthma
173c.	Occupational asthma
173d.	Work aggravated asthma
1780	Aspirin induced asthma
1781	Asthma trigger - pollen
1782	Asthma trigger - tobacco smoke
1783	Asthma trigger - warm air
1784	Asthma trigger - emotion
1785	Asthma trigger - damp
1786	Asthma trigger - animals
1787	Asthma trigger - seasonal
1788	Asthma trigger - cold air
1789	Asthma trigger respiratory inf
178A.	Asthma trigger - airborne dust
178B.	Asthma trigger - exercise
1O2..	Asthma confirmed
388t.	RCP asthma assessment
388t0	RCP asth assess 3 quest score
38DL.	Asthma control test
38DT.	Asthma control questionnaire

38DV.	Mini asthma QOL questionnaire
661M1	Asthma self-manage plan agreed
661N1	Asthma self-manage plan review
663d.	Emerg asthm adm since lst appt
663e.	Asthma restricts exercise
663e0	Asthma sometime restr exercise
663e1	Asthma severely restr exercise
663f.	Asthma never restrcts exercise
663j.	Asthma - currently active
663m.	Asth A&E attend since last vis
663n.	Asth treat compliance satisfac
663N.	Asthma disturbing sleep
663N0	Asthma causing night waking
663N1	Asthma disturbs sleep weekly
663N2	Asthma disturbs sleep freqntly
663O.	Asthma not disturbing sleep
663O0	Asthma never disturbs sleep
663p.	Asth treat compliance unsatisf
663P.	Asthma limiting activities
663P0	Asthma limit act 1-2 time mth
663P1	Asth limit activ 1 - 2 time wk
663P2	Asthma limits activit most day
663q.	Asthma daytime symptoms
663Q.	Asthma not limiting activities
663r.	Asthma night symp 1-2 per mth
663s.	Asthma never causes day symps
663t.	Asthma day symp 1-2 per mth
663u.	Asthma day symp 1-2 per week
663U.	Asthma management plan given
663v.	Asthma daytime symps most days
663V.	Asthma severity
663V0	Occasional asthma
663V1	Mild asthma
663V2	Moderate asthma
663V3	Severe asthma
663w.	Asthm limits walk hills/stairs
663x.	Asthma limits walking on flat
663y.	Num asthm exacs in past year
66Y5.	Change in asthma managemt plan
66Y9.	Step up chnge asthm managmt pl
66YA.	Step down chnge asthm manag pl
66YC.	Absent work/schl due to asthma
66YJ.	Asthma annual review
66YK.	Asthma follow-up
66Yp.	Asthma review RCP 3 questions
66YP.	Asthma night-time symptoms
66Yq.	Asthma night symptom 1 to 2 wk
66YQ.	Asthma monitoring by nurse
66Yr.	Asthma cause sympt most nights
66YR.	Asthma monitoring by doctor
66Ys.	Asthma never caus night symptm
66Yu.	Num dy abs sch asthma pst 6 mn
8793	Asthma control step 0
8794	Asthma control step 1

8795	Asthma control step 2
8796	Asthma control step 3
8797	Asthma control step 4
8798	Asthma control step 5
8B3j.	Asthma medication review
8CMA0	Pat writt asthma pers act plan
8CR0.	Asthma clin management plan
8H2P.	Emergency admission
9hA..	Except report: asthma qual ind
9hA1.	Except asthma qual ind: Pt uns
9hA2.	Excep asthma qual ind: Inf dis
9OJA.	Asthma monitoring check done
H3120	Chronic asthmatic bronchitis
H33..	Asthma
H330.	Extrinsic (atopic) asthma
H3300	Extrinsic asthma - no status
H3301	Extrinsic asthma + status
H330z	Extrinsic asthma NOS
H331.	Intrinsic asthma
H3310	Intrinsic asthma - no status
H3311	Intrinsic asthma + status
H331z	Intrinsic asthma NOS
H332.	Mixed asthma
H333.	Acute exacerbation of asthma
H334.	Brittle asthma
H335.	Chron asthm w fix airflw obstr
H33z.	Asthma unspecified
H33z0	Status asthmaticus NOS
H33z1	Asthma attack
H33z2	Late-onset asthma
H33zz	Asthma NOS
H35y6	Sequoiosis (red-cedar asthma)
H35y7	Wood asthma
H47y0	Detergent asthma

Ischaemic Heart Disease

14A3.	H/O: myocardial infarct <60
14A4.	H/O: myocardial infarct >60
14AH.	H/O: Myoc infarct in last year
14AT.	H/O: myocardial infarction
14AW.	H/O acute coronary syndrome
187..	Frequency of angina
323..	ECG: myocardial infarction
3232	ECG: old myocardial infarction
3233	ECG: antero-septal infarct.
3234	ECG:posterior/inferior infarct
3235	ECG: subendocardial infarct
3236	ECG: lateral infarction
323Z.	ECG: myocardial infarct NOS
3889	Euroscore for angina
388E.	Canad Card Soc classif angina
388F.	CLASP angina score
661M0	Angina self-manage plan agreed
661N0	Angina self-manage plan review

662K.	Angina control
662K0	Angina control - good
662K1	Angina control - poor
662K2	Angina control - improving
662K3	Angina control - worsening
662K4	Angina self manage pln commenc
662K5	Angina self manage pln complet
662Kz	Angina control NOS
662N.	CHD monitoring
6A2..	Corony heart dis annual review
6A4..	Coronary heart disease review
7920	Saphen v graft repl coronary a
79200	Saphen v graft repl 1 cor art
79201	Saphen v graft repl 2 cor art
79202	Saphen v graft repl 3 cor art
79203	Saphen v graft repl 4+ cor art
7920y	Saph vein graft repl cor a OS
7920z	Saph vein graft repl cor a NOS
7921	Other autograft rep coronary a
79210	Autograft rep 1 coronary a NEC
79211	Autograft rep 2 coronary a NEC
79212	Autograft rep 3 coronary a NEC
79213	Autogr repl 4+ coronary a NEC
7921y	Other autograft repl cor a OS
7921z	Other autograft repl cor a NOS
7922	Allograft replac coronary art
79220	Allograft replac 1 coronary a
79221	Allograft replac 2 coronary a
79222	Allograft replac 3 coronary a
79223	Allograft replac 4+ coronary a
7922y	Allograft replac coronary a OS
7922z	Allograft replac coronry a NOS
7923	Prosth replac coronary artery
79230	Prosth replac 1 coronary art
79231	Prosth replac 2 coronary art
79232	Prosth replac 3 coronary art
79233	Prosth replac 4+ coronary art
7923y	Prosth replac coronary art OS
7923z	Prosth replac coronary art NOS
7924	Revision bypass coronary art
79240	Revision bypass 1 coronary art
79241	Revision bypass 2 coronary art
79242	Revision bypass 3 coronary art
79243	Revision bypass 4 coronary art
79244	Rev conn thoracic to coronary
79245	Rev implant thorac a in heart
7924y	Revision bypass coronary a OS
7924z	Revision bypass coronary a NOS
7925	Connct mammary to coronary art
79250	Double anast mamm/coronary art
79251	Doub implant mamm/coronary art
79252	Sing anast mamm l ant desc cor
79253	Single anast mamm/coronary NEC
79254	Single implant mamm/coronary a

7925y	Connect mammary coronary a OS
7925z	Connect mammary coronary NOS
7926	Connect oth thor art coron art
79260	Double anast thor/cor art NEC
79261	Double implant thor/cor a NEC
79262	Single anast thor/cor art NEC
79263	Single implant thor/cor a NEC
7926y	Connect oth thor/coron art OS
7926z	Connect oth thor/coron art NOS
79275	Open angioplasty coronary art
7928	Translum balloon angiop coro a
79280	Percut balloon angiopl 1 cor a
79281	PC balloon angiopl mult cor ar
79282	PC balloon angiopl cor a graft
79283	Per tran cut bal angio cor art
7928y	TL balloon angiopl coron a OS
7928z	TL balloon angiopl coron a NOS
79290	PC TL laser coronary angioplas
79291	Streptok PC TL cor thrombolys
79292	PC TL inj therap sub cor a NEC
79293	Rotary coronary angioplasty
79294	Insert coronary artery stent
79295	Insert drug-elut cor art stent
79296	Percut translum ather cor art
792B0	Endarterectomy coronary a NEC
792D.	Other bypass coronary artery
792Dy	Other bypass coronary art OS
792Dz	Other bypass coronary art NOS
792E.	Percutan coronary interventn
7.92E+02	Emrgncy percut coronry intrvnt
793G.	Perc tran bal ang sten cor art
793G0	P t ba an in 1-2 dr el st co a
793G1	P t b an in 3 mo dr el st co a
793G2	Pe tr bal an in 1-2 ste co art
793G3	Pe co ba ang 3 mo st co ar NEC
793Gy	OS per tra bal ang ste cor art
793Gz	Per tra bal ang ste cor ar NOS
8B27.	Antianginal therapy
8B3k.	CHD medication review
8CMP.	Coronry hart disease care plan
8H2V.	Admit isch heart dis emergency
8IEY.	Rf Ang Pln slf-mgt prg decld
8T04.	Ref Angina Plan self-mgt prg
9Ob0.	Attends CHD monitoring
9Ob1.	Refuses CHD monitoring
9Ob8.	CHD monitoring check done
G3...	Ischaemic heart disease
G30..	Acute myocardial infarction
G300.	Acute anterolateral infarction
G301.	Anterior myocard. infarct OS
G3010	Acute anteroapical infarction
G3011	Acute anteroseptal infarction
G301z	Anterior myocard.infarct NOS
G302.	Acute inferolateral infarction

G303.	Acute inferoposterior infarct
G304.	Posterior myocard.infarct NOS
G305.	Lateral myocardial infarct NOS
G306.	True posterior myocard.infarct
G307.	Acute subendocardial infarct
G3070	Acute non-Q wave infarction
G3071	Acute non-ST seg elevation mi
G308.	Inferior myocard. infarct NOS
G309.	Acute Q-wave infarct
G30B.	Acute posterol myocard infarct
G30X.	Ac transmur MI unspec site
G30X0	Acute ST segment elevation mi
G30y.	Other acute myocardial infarct
G30y0	Acute atrial infarction
G30y1	Acute papillary muscle infarct
G30y2	Acute septal infarction
G30yz	Other acute myocardial inf.NOS
G30z.	Acute myocardial infarct. NOS
G31..	Other acute/subacute IHD
G311.	Preinfarction syndrome
G3110	Myocardial infarction aborted
G3111	Unstable angina
G3112	Angina at rest
G3113	Refractory angina
G3114	Worsening angina
G3115	Acute coronary syndrome
G311z	Preinfarction syndrome NOS
G312.	Coron thromb/not result in MI
G31y.	Other acute/subacute IHD
G31y0	Acute coronary insufficiency
G31y1	Microinfarction of heart
G31y2	Subendocardial ischaemia
G31y3	Transient myocardial ischaemia
G31yz	Other acute/subacute IHD NOS
G32..	Old myocardial infarction
G33..	Angina pectoris
G330.	Angina decubitus
G3300	Nocturnal angina
G330z	Angina decubitus NOS
G33z.	Angina pectoris NOS
G33z0	Status anginosus
G33z1	Stenocardia
G33z2	Syncope anginosa
G33z3	Angina on effort
G33z4	Ischaemic chest pain
G33z5	Post infarct angina
G33z6	New onset angina
G33z7	Stable angina
G33zz	Angina pectoris NOS
G34..	Other chr.ischaemic heart dis.
G340.	Coronary atherosclerosis
G3400	Single coronary vessel disease
G3401	Double coronary vessel disease
G342.	Atherosclerotic cardiovasc dis

G343.	Ischaemic cardiomyopathy
G344.	Silent myocardial ischaemia
G34y.	Other specif. chronic IHD
G34y0	Chronic coronary insufficiency
G34y1	Chronic myocardial ischaemia
G34yz	Other specif.chronic IHD NOS
G34z.	Other chronic IHD NOS
G34z0	Asymptomatic CHD
G35..	Subseqnt myocardial infarction
G350.	Subsqnt myocrd infarc/ant wall
G351.	Subsqnt myocrd infarc/inf wall
G353.	Subseq myo infarct other sites
G35X.	Subseq MI of unspec site
G36..	Certain curnt comp fol acut MI
G360.	Haempericrd/cur comp fol ac MI
G361.	Atrl sept def/c comp fol ac MI
G362.	Vent sep def/c comp fol ac MI
G363.	Rup cd w w't h'mpc/cmp f ac MI
G364.	Rp chord tend/c cmp fol ac MI
G365.	Rp papilr musc/c cmp fol ac MI
G366.	Thrm/atr
G38..	Postoperative MI
G380.	Postop transmur MI inf wall
G381.	Postop transm MI inferior wall
G382.	Postop transm MI other sites
G383.	Postop transm MI unspec site
G384.	Postop subendocardial MI
G38z.	Postop MI
G39..	Coronary microvascular disease
G3y..	Ischaemic heart disease OS
G3z..	Ischaemic heart disease NOS
G501.	Post infarction pericarditis
Gyu3.	[X]Ischaemic heart diseases
Gyu30	[X]Other forms/angina pectoris
Gyu31	[X]Oth currnt comp follw ac MI
Gyu32	[X]Oth form/ac ischaem hrt dis
Gyu33	[X]O form/chron ischmc hrt dis
Gyu34	[X]Ac transmur MI unspec site
Gyu35	[X]Subseq myocard inf oth site
Gyu36	[X]Subseq MI of unspec site
SP076	Coronary art bypass graft occl
ZV457	[V]Pres/aortcoronry bypss grft
ZV458	[V]Pres/coron angiopl impl+gft
ZV45K	[V]Pres coron art bypass graft
ZV45L	[V]Stat foll coron angiopl NOS

Pulmonary Fibrosis	
23E5.	O/E - fibrosis of lung present
A114.	Tuberculous fibrosis of lung
H41..	Asbestosis
H41z.	Asbestosis NOS
H423.	Massive silicotic fibrosis
H431.	Bauxite fibrosis of lung
H433.	Graphite fibrosis of lung

H4642	Chemical pulmonary fibrosis
H48..	Progressive massive fibrosis
H4y1.	Chronic pulm.radiation disease
H4y10	Radiation pulmonary fibrosis
H4y1z	Chronic pulm.radiation dis.NOS
H4y21	Chr drg-indc interst lung diso
H55..	Postinflammatory pulm.fibrosis
H563.	Idiopath.fibrosing alveolitis
H5631	Diffuse pulmonary fibrosis
H5632	Pulmonary fibrosis
H5633	Usual interstitial pneumonitis
H563z	Idiopath.fibrosing alveol.NOS
h8M..	PIRFENIDONE
h8M1.	ESBRIET 267mg capsules
h8M2.	PIRFENIDONE 267mg capsules

Clinical variables

Baseline demographic and clinical characteristics of the COPD cohort are shown in table 1 of the main manuscript. Read Code lists are provided for BMI, FEV1, FEV1 %predicted, FEV1/FVC %, FVC, smoking status and specified respiratory medications. Read codes are also listed for MRC breathlessness grades 1 to 5.

Body Mass Index

22K..	Body Mass Index
22K1.	Body Mass Index normal K/M2
22K2.	Body Mass Index high K/M2
22K3.	Body Mass Index low K/M2
22K4.	BMI 25-29 - overweight
22K5.	Body mass index 30+ - obesity
22K6.	Body mass index less than 20
22K7.	BMI 40+ - severely obese
22K8.	Body mass index 20-24 - normal
22K9.	Body mass index centile
22K90	Baseline BMI centile
22KB.	Baseline body mass index

Categories of BMI

Underweight	BMI value < 18.5
Normal range	18.5 ≥ BMI value < 25
Overweight	25 ≥ BMI value < 30
Obese	30 ≥ BMI value < 35
Very obese	BMI value ≥ 35

FEV1 Assessment

339a.	FEV1 before bronchodilation
339b.	FEV1 after bronchodilation
339e.	FEV1 pre steroids
339f.	FEV1 post steroids
339O.	Forced expired volume in 1 sec

FEV1 %predicted

339S.	Percent predicted FEV1
339S0	Percent pred FEV1 bronchodiln

FEV1/FVC %

3398	FEV1/FVC ratio normal
3399	FEV1/FVC ratio abnormal
339j.	FEV1/FVC ratio pre steroids
339k.	FEV1/FVC ratio post steroids
339l.	FEV1/FVC ratio befr bronchodil
339m.	FEV1/FVC ratio after bronchdil
339M.	FEV1/FVC ratio
339R.	FEV1/FVC percent
339T.	FEV1/FVC > 70% of predicted
339U.	FEV1/FVC < 70% of predicted

FVC Assessment

3396.	Forced vital capacity - FVC
33960	Forced vital capacity normal
33961	Forced vital capacity abnormal
33963	FVC after chng bronchodilator
339h.	FVC after bronchodilation
339h0	FVC post bronchodi percnt chng
339s.	FVC before bronchodilation

Smoking status

Read code lists for smoking status were created by a GP researcher (LJ) familiar with Read code use in clinical practice, using a combined technique of Read Term “string searches” and searches through the hierarchy of parent Read codes.

In defining active smokers, we included those smoking cessation advice codes (including codes for medication used in smoking cessation support) which implied that the patient was an active smoker at that time. We especially *excluded* those few non-specific smoking advice codes (that could equally be employed in counselling never smokers as smokers) from any of the codes that defined our smoking categories. Our ex-smoker code list includes only codes that indicate that a patient has stopped smoking at the time of the record. We believe that we reduced the likelihood of misclassifying patients in the process of giving up smoking as “ex-smokers” by including all smoking-cessation support codes in the list of codes that define “active smokers”. We have quantified the time, in months, between this last smoking code and the start of the observation period, and confirmed that these defining codes are recent.

Never smokers were reclassified as ex-smokers if there were contradictory preceding smoking codes that implied a past smoking history; patients in whom smoking status had been reassigned in this way were “flagged” so that this process could be identified. An uncommon problem particular to electronic records is that, sporadically, multiple conflicting codes are recorded on the same day. In order to avoid bias, we determined prior to data analysis that if smoking status codes for both active and ex-smoker appeared on the same date, one code would be selected at random.

Active Smoker

1372	Trivial smoker - < 1 cig/day
1373	Light smoker - 1-9 cigs/day
1374	Moderate smoker - 10-19 cigs/d
1375	Heavy smoker - 20-39 cigs/day
1376	Very heavy smoker - 40+cigs/d
137..	Tobacco consumption

137b.	Ready to stop smoking
137c.	Thinking about stop smoking
137C.	Keeps trying to stop smoking
137d.	Not interested in stop smoking
137D.	Admitted tobacco cons untrue?
137e.	Smoking restarted
137E.	Tobacco consumption unknown
137f.	Reason for restarting smoking
137G.	Trying to give up smoking
137h.	Min from wake to 1st tobac con
137H.	Pipe smoker
137J.	Cigar smoker
137m.	Failed attempt to stop smoking
137M.	Rolls own cigarettes
137n.	Total time smoked
137P.	Cigarette smoker
137Q.	Smoking started
137R.	Current smoker
137V.	Smoking reduced
13p0.	Negotiatd date cessatn smoking
13p5.	Smoking cessn progm start date
13p50	Practice smok cess pr strt dat
67H6.	Brf intervention smoking cessn
745H.	Smoking cessation therapy
745H0	Nic rep thera us nicot ptches
745H1	Nicot repl thera us nicot gum
745H2	Nic repl thera us nic inhalatr
745H3	Nic repl thera us nic lozenges
745H4	Smoking cessation drug therapy
745Hy	OS smoking cessation therapy
745Hz	Smoking cessation therapy NOS
8CAg.	Smoke cess advi prov com pharm
8CAL.	Smoking cessation advice
8CdB.	Stop smok serv opport signpost
8H7i.	Referral: smok cessatn advisor
8HBM.	Stop smoking fce to fce flw-up
8HBP.	Smoking cesn 12 week follow-up
8HkQ.	Refer to NHS stop smoking srvc
8HTK.	Referl to stop-smoking clinic
8IAj.	Smok cessation advice declined
8IEK.	Smok cessation program declned
8IEM.	Smoking cess drug therapy decl
8IEo.	Ref smoking cessn serv declnd
8T08.	Ref to smoking cessn service
9hG..	Except repor: smoking qual ind
9hG0.	Except smok qual ind: Pt uns
9hG1.	Except smok qual ind: Inf diss
9kc..	Smoking cessation - enh se adm
9kc0.	Smok ces templt completd - ESA
9kf1.	Ref COPD stru smok asses - ESA
9kf2.	COPD stru smok ass decld - ESA
9ko..	Currnt smokr annua reviw - ESA
9N2k.	Seen by smoking cesstn advisor
9N4M.	DNA - Smoking cessation clinic

9Ndg.	Dec con f flw up by smk cess t
9NdZ.	Dec con for smok cess data sha
9NS02	Ref smoking cessat serv offer
9OO..	Stop smoking monitoring admin.
9OO1.	Attends stop smoking monitor.
9OO2.	Refuses stop smoking monitor
9OO3.	Stop smoking monitor default
9OO4.	Stop smoking monitor 1st lettr
9OO5.	Stop smoking monitor 2nd lettr
9OO6.	Stop smoking monitor 3rd lettr
9OO7.	Stop smoking monitor verb.inv.
9OO8.	Stop smoking monitor phone inv
9OO9.	Stop smoking monitoring delete
9OOA.	Stop smoking monitor.chck done
9OOB.	Stop smokg invtn SMS txt mssge
9OOB0	Stop smoking ivtn 1st txt msge
9OOB1	Stop smoking ivtn 2nd txt msge
9OOB2	Stop smoking ivtn 3rd txt msge
9OOZ.	Stop smoking monitor admin.NOS

Ex-Smoker

1377	Ex-trivial smoker (<1/day)
1378	Ex-light smoker (1-9/day)
1379	Ex-moderate smoker (10-19/day)
137A.	Ex-heavy smoker (20-39/day)
137B.	Ex-very heavy smoker (40+/day)
137F.	Ex-smoker - amount unknown
137j.	Ex-cigarette smoker
137K.	Stopped smoking
137K0	Recently stopped smoking
137l.	Ex roll-up cigarette smoker
137L.	Current non-smoker
137N.	Ex pipe smoker
137O.	Ex cigar smoker
137S.	Ex smoker
137T.	Date ceased smoking
13p4.	Smoking free weeks
9km..	Ex-smoker annual review - ESA

Never Smoker

1371	Never smoked tobacco

MRC breathlessness scale (Medical Research Council, 1986)

173H.	MRC Breathless Scale: grade 1
173I.	MRC Breathless Scale: grade 2
173J.	MRC Breathless Scale: grade 3
173K.	MRC Breathless Scale: grade 4
173L.	MRC Breathless Scale: grade 5

Respiratory Medication

Read code lists and equivalent EMIS codes (customised codes used by practices that employ EMIS software) are shown for the 5 groups of inhaled medications: short-acting beta-agonists (SABA), short-acting antimuscarinic bronchodilators (SAMA), long acting beta-agonists (LABA), long acting antimuscarinic bronchodilators (LAMA) and inhaled corticosteroids (ICS). Combination inhalers appear in each group in which their constituents belong.

SABA

Salbutamol inhaler

EADR19043NEMIS, EADR19044NEMIS, c1E7., c1E8., PUDR11413NEMIS, c1E2., SAAC28242EMIS, SAAE6755, SAAU28629EMIS, SABR14648NEMIS, SABR15868NEMIS, SABR26803EMIS, SABR31775EMIS, SABR35182EMIS, SACF26553EMIS, SACY24562EMIS, SACY24563EMIS, SADI19640EMIS, SADI19641EMIS, SADR1866NEMIS, SADR1730NEMIS, SADR1768NEMIS, SADR1769NEMIS, SADR1786NEMIS, SADR1787NEMIS, SADR1788NEMIS, SADR1789NEMIS, SADR5080NEMIS, SADR21103NEMIS, SADR21104NEMIS, SAIN29844NEMIS, SAIN29845NEMIS, SAIN2483, SAIN31771EMIS, SARE19642EMIS, SARE19643EMIS, SARO2488, SARO2489, ASP28840EMIS, c13J., c13G., c13N., c13P., c13v., c13U., c13R., c1EA., c1EC., c13q., SADR12726NEMIS, c13C., c13x., c13L., c13E., c13r., SADR12728NEMIS, c13D., c13y., c13F., c13S., SAIN8676WTKSN, SACF3932NEMIS, SAIN23709EMIS, c13Z., c13H., SABR26801EMIS, SACF10856NEMIS, c13K., c1E1., AIIN26490EMIS, c13I., c13V., AIBR35356EMIS, c13Q., ASBR31773EMIS, ASIN31769EMIS, PUDR11413NEMIS, c1E2., NOIN29847NEMIS, NOIN29848NEMIS, SACF34NEMIS, SAIN2479, c13Y., c133., SAIN40204NEMIS, SAIN40203NEMIS, c1E9., c1EB., SAIN40204NEMIS, VEAC28240EMIS, VEIN3026, VERO3031, VERO3032, c134., c13T., c13M., c136., c137., VEBR31124EMIS, c13O., VECF34087EMIS

Fenoterol inhaler

c154., c15y., FEIN9113EMIS, FEIN4913, FEAE10342BRIDL

Terbutaline inhaler

c14f., c14j.

Salbutamol nebulised

SANE2486, SANE9041BRIDL, SANE10644BRIDL, SARE2487, c13z., c13w., SANE48179NEMIS, c1EE., c13o., SANE48180NEMIS, SANE48179NEMIS, SANE48180NEMIS, SANE20381EMIS, STNE22543EMIS, STNE22544EMIS, c13A., c13B., DEGRADE_VENE3029, VENE3029, VENE8699BRID, VERE3030, c139., c135., c1ED., c13m.

Fenoterol nebulised:

c15z., FESO4914

Terbutaline nebulised

c147., c14w.

SAMA

Ipratropium alone

c31u., c31x., c31z., IPAU23857EMIS, IPCF17452NEMIS, IPDR25769EMIS, IPDR25770EMIS, IPAE10449BRIDL, IPAE10447BRIDL, c31t., c31A., c31B., ATAE25765EMIS, ATAE25766EMIS, ATAE246, ATAU23855EMIS, ATCF17453NEMIS, c315., c311., c31G., c318., c319., ATIN248, c313.

Oxitropium inhaler

c324., c321., OXAU23864EMIS, OXIN9076EMIS, OXAU23862EMIS, OXIN9078EMIS, c323., c322.

Ipratropium nebulised

c31v., c31y., c31w., IPNE36455NEMIS, IPNE1494, IPNE35204NEMIS, IPNE9128BRIDL, IPNE35204NEMIS, IPNE24828EMIS, IPNE24829EMIS, IPNE30103NEMIS, IPNE36455NEMIS, c316., c317., ATNE8769BRIDL, ATNE247, ATNE35205NEMIS, ATNE9126BRIDL, c314., ATNE35205NEMIS, c312., c31C., c31D., RENE32712EMIS, RENE32713EMIS

SAMA/SCG

Salbutamol/Sodium Cromoglycate inhaler

c72y., c72z.

SABA/SAMA combined

Salbutamol/Ipratropium inhaler

IPIN18579NEMIS, c51C., c51D., COIN24315EMIS

Fenoterol/Ipratropium inhaler

c51A., c51B., FEAE1179NEMIS

Salbutamol/Ipratropium nebulised:

IPNE36773NEMIS, IPNE18580NEMIS, c51H., c51F., c51E., COUN26806EMIS, COIN626NEMIS

Fenoterol/Ipratropium nebulised

FENE18581NEMIS, c51w.

LABA

Formoterol (alone)

'EFBR31957EMIS','EFBR31958EMIS','EFDR28163EMIS','FODR17644NEMIS','FODR24335NEMIS','FODR24334NEMIS','FODR17643NEMIS','FOIN17645NEMIS','FOPR21152NEMIS','FODR24340NEMIS','c1C8.','c1C1.','c1Cz.','c1C4.','c1Cy.','c1C3.','ATPR21154NEMIS','c1C7.','FODR28161EMIS','c1C2.','c1C6.','c1C5.','OXBR31953EMIS','OXBR31954EMIS',

Formoterol (combination inhaler): with beclometasone

'BECF28854NEMIS','c6Az.', FOCF28856NEMIS,'c6A1.',

Formoterol (combination inhaler): with budesonide

'BUDR18591NEMIS','BUDR18592NEMIS','BUDR18593NEMIS','c67z.','c67y.','c67x.','SYTU10847NEMIS','SYTU10848NEMIS','SYTU15234NEMIS','c671.','c672.','c673.',

Formoterol (combination inhaler): with fluticasone

'FLCF76573NEMIS','FLCF76574NEMIS','FLCF76572NEMIS','FLCF76573NEMIS','FLCF76574NEMIS','FLCF76572NEMIS','c1cy.','c1cx.','c1cz.','FLCF76577NEMIS','FLCF76578NEMIS','FLCF76576NEMIS','c1c2.','FLCF76577NEMIS','c1c3.','FLCF76578NEMIS','c1c1.','FLCF76576NEMIS',

Salmeterol (alone)

'c191.','c19z.','c195.','c197.','c196.','SAAC26709EMIS','SAAE9073EMIS','SACF21617NEMIS','SADI10715BRIDL','SADR1873NEMIS','SADR1874NEMIS','SARE9074EMIS','SEAC26707EMIS','SEAE10636BRIDL','SEDI8939BRECO','SERE8940BRECO','c199.','c192.','c198.','c193.','c194.','SECF21619NEMIS',

Salmeterol (combination inhaler) with fluticasone

'FLCF18584NEMIS','FLCF18585NEMIS','FLCF18583NEMIS','FLDR18586NEMIS','FLDR18587NEMIS','FLDR18588NEMIS','c1Dv.','c1Dw.','c1Du.','c1Dx.','c1Dy.','c1Dz.','c1D1.','SEDR35415EMIS','c1D5.','SECF4758NEMIS','c1D2.','c1D6.','SEDR35418EMIS','SECF4760NEMIS','c1D4.','SECF4756NEMIS','c1D3.','SEDR35421EMIS',

Vilanterol (combination inhaler): with fluticasone

'c6B4.','FLDR86732NEMIS','FLDR86734NEMIS','FLDR86734NEMIS','REDR86737NEMIS','REDR86736NEMIS','c6B1.','c6B1.','c6B1.','c6B3.','REDR86737NEMIS','c6B3.','c6B3.',

Indacaterol (alone)

'ININ43604NEMIS','ININ43606NEMIS','c1b2.','ININ43604NEMIS','c1b4.','ININ43606NEMIS','ONIN43608NEMIS','ONIN43609NEMIS','c1b1.','ONIN43608NEMIS','c1b3.','ONIN43609NEMIS',

LAMA

Tiotropium (alone)

'TIDR13729NEMIS','TIDR13727NEMIS','TISO27048NEMIS','c331.','c332.','c333.','c33z.','SPDR13730NEMIS','c33y.','SPDR13731NEMIS','SPSO27050NEMIS','c33x.',

Aclidinium (alone)

'c342.','ACDR76091NEMIS','ACDR76091NEMIS','EKDR76095NEMIS','c341.',

Glycopyrronium (alone)

'GLIN77079NEMIS','GLIN77079NEMIS','o324.','o324.','o323.','o324.','o323.','SEIN77082NEMIS','SEIN77082NEMIS',

ICS

Beclametasone dipropionate (alone)

'BEBR17528NEMIS','BEBR17526NEMIS','BEBR17527NEMIS','BEBR17551NEMIS','BEBR17550NEMIS','BECF17549NEMIS','BECF22845NEMIS','BECF22846NEMIS','BECF17548NEMIS','BEDR17541NEMIS','BEDR17542NEMIS','BEDR17540NEMIS','BEDR17531NEMIS','BEDR17532NEMIS','BEDR17533NEMIS','BEDR17544NEMIS','BEDR17547NEMIS','BEDR17545NEMIS','BEDR17546NEMIS','BEDR17543NEMIS','BEIN17538NEMIS','BEIN17537NEMIS','BEIN17525NEMIS','BEIN17539NEMIS','BERE17534NEMIS','BERE17535NEMIS','BERE17536NEMIS','c66L.','BEDR12714NEMIS','c66M.','BEDR12716NEMIS','c66N.','BEDR12718NEMIS','c61p.','c61w.','c61F.','c61z.','c66B.','c66W.','c66Y.','c61r.','c61q.','c61x.','c61u.','c66H.','c61s.','c66g.','c61E.','c61t.','c66C.','c66h.','c61A.','c61h.','c66G.','c61B.','c61D.','c61v.','c66A.','c66X.','c66V.','BEAU17921EMIS','BEAU17922EMIS','BEBR14402NEMIS','BEBR14400NEMIS','BEBR14401NEMIS','BEBR14399NEMIS','BEBR14398NEMIS','BEBR1868NEMIS','BEBR1872NEMIS','BEBR1867NEMIS','BEBR26471EMIS','BEBR26472EMIS','BEBR26473EMIS','BEDI19630EMIS','BEDI19631EMIS','BEDI19632EMIS','BEDR1732NEMIS','BEDR1733NEMIS','BEDR1731NEMIS','BEDR1771NEMIS','BEDR1772NEMIS','BEDR1770NEMIS','BEDR1869NEMIS','BEDR1870NEMIS','BEDR1871NEMIS','BEDR5082NEMIS','BEDR5083NEMIS','BEDR5081NEMIS','BEDR11414NEMIS','BEDR11415NEMIS','BEDR11416NEMIS','BEIN280','BEIN18072EMIS','BEAE4626','BEIN281','BEIN26593EMIS','BEIN31782EMIS','BEIN32627EMIS','BEIN31783EMIS','BEPR14397NEMIS','BEPR14396NEMIS','BERE26594EMIS','BERE19633EMIS','BERE19634EMIS','BERE19635EMIS','BERO284','BERO285','BERO10442BRIDL','BESP28850EMIS','BESP28851EMIS','BESP28852EMIS','BESU286','c61X.','c61C.','c61S.','c61Z.','c61T.','c61V.','BEIN8671WTKSN','EADR21092NEMIS','c66b.','PUDR11418NEMIS','PUDR11419NEMIS','PUDR11420NEMIS','c66I.','c66J.',c66K.'PUDR11418NEMIS','PUDR11419NEMIS','PUDR11420NEMIS','c66I.','c66J.','c66K.','c66E.','c66F.','c66D.','ASBR34121EMIS','ASBR34122EMIS','ASBR34123EMIS','ASIN31778EMIS','ASIN31779EMIS','ASIN32625EMIS','CLCF22851NEMIS','CLCF22852NEMIS','CLCF22854NEMIS','CLCF22848NEMIS','c66d.','c66e.','c66f.','c66c.','c619.','c66P.','c61c.','c66S.','c61a.','c66Q.','c61d.','c66T.','c61e.','c66R.','c61f.','c66U.','BEDI8397EGTON','BEDI8398EGTON','BEDI9501BRIDL','BERE8399EGTON','BERE8400EGTON','BERE9503BRIDL','BEPO8976BRIDL','BEPO8974BRIDL','c668.','QVCF33772EMIS','c666.','QVCF33776EMIS','c667.','QVCF33770EMIS','c665.','QVCF33774EMIS','QVBR19056NEMIS','QVBR19055NEMIS','c66a.','c66Z.',

Beclametasone dipropionate (combination inhaler): with formoterol

'BECF28854NEMIS','c6Az.','FOCF28856NEMIS','c6A1.',

Budesonide (alone)

'BUAE4665','BUAE4666','BUBR34628EMIS','BUBR34629EMIS','BUBR34630EMIS','BUCF33454NEMIS','BUCF33455NEMIS','BUDR12719NEMIS','BUDR12720NEMIS','BUDR21820NEMIS','BUDR21821NEMIS','BUDR21822NEMIS','BUIN18278NEMIS','BUIN19789NEMIS','BUIN13799NEMIS','BURE18821EMIS','BURE18822EMIS','BUSP8941DLSEM','BUSP8939DLSEM','BUTU19623EMIS','BUTU10005BRIDL','BUTU10007BRIDL','c64d.','c64L.','c64k.','BUDR12722NEMIS','c64m.','c64g.','c64v.','c64A.','c64z.','c64N.','c64u.','c64o.','c64F.','c64l.','BUDR12724NEMIS','c64n.','c64h.','c64y.','c64e.','c64B.','EADR21824NEMIS','EADR21825NEMIS','EADR21826NEMIS','c64H.','c64I.','c64J.','NOIN18280NEMIS','NOIN19790NEMIS','c64p.','c64G.','PUAE4237','PUCF33456NEMIS','PUCF33457NEMIS','PURE8390BRID','PUSP8944DLSEM','PUSP8945DLSEM','DEGRADE_PUTU19548EMI','PUTU19548EMIS','PUTU10001BRIDL','PUTU10003BRIDL','c64c.','c64K.','c641.','c647.','c642.','c643.','c64C.','c648.','c64M.','c64E.','c649.','PUIN13798NEMIS','PUAE4238','PURE9247BRIDL','c644.','c645.','c64D.','PUBR34622EMIS','PUBR34623EMIS','PUBR34624EMIS',

Budesonide (combination inhaler) with formoterol

'BUDR18591NEMIS','BUDR18592NEMIS','BUDR18593NEMIS','c67z.','c67y.','c67x.','SYTU10847NEMIS','SYTU10848NEMIS','SYTU15234NEMIS','c671.','c672.','c673.',

Ciclesonide (alone)

'CICF33575NEMIS','CICF19411NEMIS','CICF19951NEMIS','CICF19409NEMIS','c69z.','c69y.','ALCF33576NEMIS','ALCF19414NEMIS','ALCF19952NEMIS','ALCF19413NEMIS','c691.','c692.',

Fluticasone propionate (alone)

'c655.','c65Q.','c65B.','c65I.','c656.','c65J.','c65R.','c65C.','c65G.','c65N.','c65S.','c65O.','c654.','c65H.','c65P.','c65A.','FLAC26702EMIS','FLAC26703EMIS','FLAC26704EMIS','FLAC26705EMIS','FLCF3683NEMIS','FLCF3684NEMIS','FLCF4774NEMIS','FLDI22584EMIS','FLDI22585EMIS','FLDI22586EMIS','FLDI24085EMIS','FLDR1876NEMIS','FLDR1877NEMIS','FLDR1875NEMIS','FLDR1878NEMIS','FLDR1880NEMIS','FLDR1881NEMIS','FLDR1879NEMIS','FLDR1882NEMIS','FLIN23891EMIS','FLIN23892EMIS','FLIN24086EMIS','FLIN23893EMIS','FLRE22587EMIS','FLRE22588EMIS','FLRE22589EMIS','FLRE24087EMIS','c65b.','c65c.','c65f.','FLAC26694EMIS','FLAC26695EMIS','FLAC26696EMIS','FLAC26697EMIS','FLDI22572EMIS','FLDI22573EMIS','FLDI22574EMIS','FLDI24079EMIS','FLIN23885EMIS','FLIN23886EMIS','FLIN24080EMIS','FLIN23887EMIS','FLRE22575EMIS','FLRE22576EMIS','FLRE22577EMIS','FLRE24081EMIS','c652.','c65U.','c658.','c65d.','c65F.','c653.','c65V.','c65e.','c65K.','c659.','c65D.','c65L.','c65W.','c65M.','c65T.','c651.','c65g.','c65E.','c657.','FLCF3681NEMIS','FLCF3682NEMIS','FLIN4775NEMIS',

Fluticasone propionate (combination inhaler): with formoterol

'FLCF76572NEMIS','FLCF76573NEMIS','FLCF76574NEMIS','FLCF76572NEMIS','FLCF76573NEMIS','FLCF76574NEMIS','c1cy.','c1cx.','c1cz.',
'FLCF76577NEMIS','FLCF76578NEMIS','FLCF76576NEMIS','c1c2.','FLCF76577NEMIS','c1c3.','FLCF76578NEMIS','c1c1.','FLCF76576NEMIS',

Fluticasone propionate (combination inhaler): with salmeterol

'FLCF18584NEMIS','FLCF18585NEMIS','FLCF18583NEMIS','FLDR18586NEMIS','FLDR18587NEMIS','FLDR18588NEMIS','c1Dv.','c1Dw.','c1Du.','c1Dx.','c1Dy.','c1Dz.',
'c1D1.','SEDR35415EMIS','c1D5.','SECF4758NEMIS','c1D2.','c1D6.','SEDR35418EMIS','SECF4760NEMIS','c1D4.','SECF4756NEMIS','c1D3.','SEDR35421EMIS',

Fluticasone propionate (combination inhaler): with vilanterol

c6B4., 'REDR86737NEMIS','REDR86736NEMIS','c6B1.','c6B1.','c6B1.','c6B3.','REDR86737NEMIS','c6B3.','c6B3.',

Mometasone furoate (alone): mometasone furoate

'MODR44710NEMIS','MODR44711NEMIS','MOMU14784NEMIS','MOMU14786NEMIS','MOMU14787NEMIS','MOMU14785NEMIS','c681.','c682.','ASDR44712NEMIS','ASDR44712NEMIS','ASMU14789NEMIS','ASMU14790NEMIS','ASMU14791NEMIS','ASMU14792NEMIS','c683.','c684.',

Supplementary Table 1. Predictors of absent AFO versus variable or persistent AFO[a]

		Odds ratio (OR)[b]	95% confidence interval	P-value	
Age (per year)		0.973	(0.966, 0.979)	<0.001	
Gender: Male		0.706	(0.621, 0.803)	<0.001	
Smoking status					
Current smoker (reference)		1.000			
Ex-smoker		1.219	(1.061, 1.399)	0.005	
Never smoker		3.425	(2.391, 4.906)	<0.001	
Body mass index, kg/m^2					
≥18.5 and <25 (reference)		1.000			
<18.5		1.056	(0.706, 1.579)	0.792	
≥25 and <30		1.564	(1.325, 1.847)	<0.001	
≥30 and <35		2.014	(1.678, 2.418)	<0.001	
≥35		2.474	(2.008, 3.050)	<0.001	
Individual comorbidities					
Anxiety/Depression		1.224	(1.078, 1.391)	0.002	
Asthma		0.817	(0.722, 0.924)	0.001	
Bronchiectasis		1.187	(0.891, 1.582)	0.241	
Cerebrovascular Disease		1.266	(1.021, 1.570)	0.031	
Chronic Kidney Disease		1.313	(1.100, 1.566)	0.003	
Connective Tissue Disease		1.046	(0.722, 1.516)	0.810	
Dementia		1.618	(0.982, 2.665)	0.059	
Diabetes		1.323	(1.121, 1.560)	0.001	
Gastro-oesophageal Reflux		1.308	(1.102, 1.552)	0.002	
Heart Failure		1.191	(0.916, 1.550)	0.191	
Hyperlipidaemia		1.264	(1.088, 1.467)	0.002	
Hypertension		0.994	(0.870, 1.135)	0.926	
Ischaemic Heart Disease		1.112	(0.942, 1.313)	0.208	
Lung Cancer		0.315	(0.076, 1.303)	0.111	
Obstructive Sleep Apnoea		1.005	(0.621, 1.627)	0.982	
Osteoporosis		0.944	(0.726, 1.229)	0.670	
Peripheral Vascular Disease		0.772	(0.569, 1.046)	0.095	
Pulmonary Fibrosis		1.835	(1.150, 2.930)	0.011	
Rhinosinusitis		1.191	(1.016, 1.394)	0.031	

[a]Using multivariable logistic regression where the outcome is the odds of absent AFO versus persistent/variable AFO, and all values of predictor variables are as at baseline.
[b]Estimated for N=11331 subjects having values observed for all the variables used in this multivariable regression model.

Supplementary Figure 1. Distribution of body mass index (BMI) by evidence of airflow obstruction since COPD diagnosis. BMI is categorised according to World Health Organisation standards.

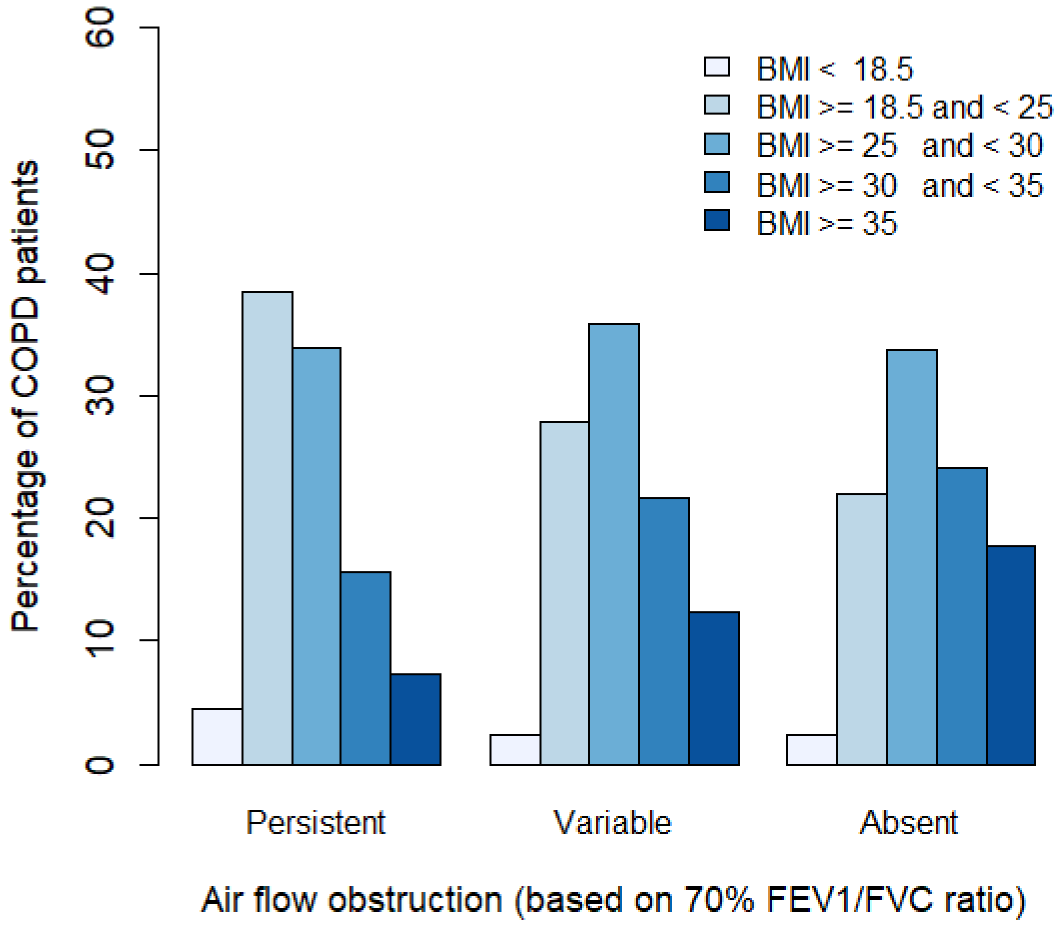

Supplementary Figure 2. Distribution of MRC breathlessness scores by evidence of airflow obstruction since COPD diagnosis.

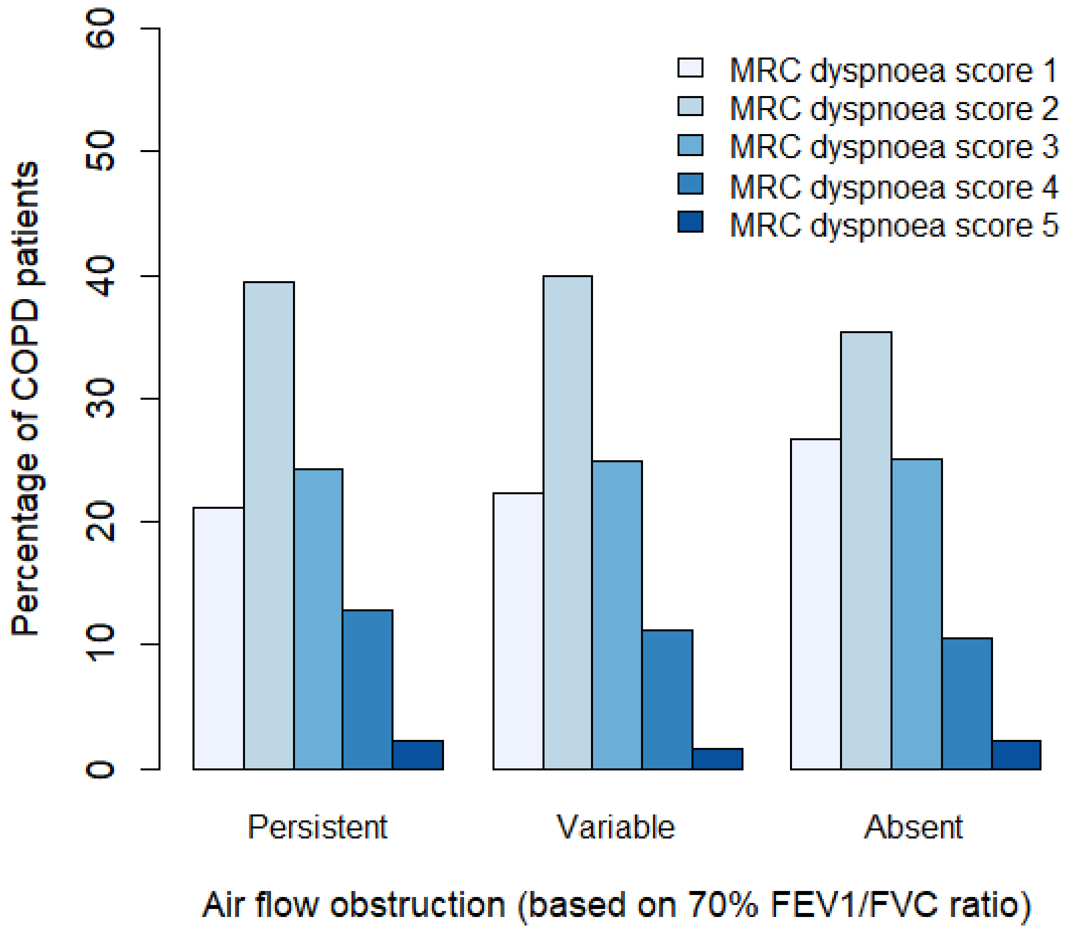

Supplementary References

1. Care and Health Information Exchange (CHIE). https//www.chie.org.uk (formerly Hampshire Health Record, http://www.hantshealthrecord.nhs.uk) Updated 2018. Date last accessed July 9th 2018.
2. Department for Communities and Local Government. *The English Indices of Deprivation Statistical Release 2010.* https://www.gov.uk/government/statistics/english-indices-of-deprivation-2010. Published March 24th 2011. Date last accessed July 9th 2018.
3. Quanjer, P. H., Tammeling, G. J., Cotes, J. E., Pedersen, O. F., Peslin, R., & Yernault, J. C. *Lung volumes and forced ventilatory flows. Official statement of the European Respiratory Society.* Eur Respir J 6.Suppl 16 (1993): 5-40.
4. Health and Social Care Information Centre. *Technology Reference data Update Distribution Service (TRUD).* https://isd.hscic.gov.uk. Date last updated June 2nd 2016. Date last accessed July 9th 2018.
5. Quality outcome framework (QoF) business rules version 25. http://www.pcc-cic.org.uk/article/qof-business-rules-v25.0 . Date last updated April 3rd 2013. Date last accessed July 9th 2018.

Supplementary Table 2. The RECORD statement – checklist of items, extended from the STROBE statement, that should be reported in observational studies using routinely collected health data.

	Item No.	STROBE items	Location in manuscript where items are reported	RECORD items	Location in manuscript where items are reported
**Title and abstract**					
	1	(a) Indicate the study's design with a commonly used term in the title or the abstract.<br><br>(b) Provide in the abstract an informative and balanced summary of what was done and what was found.	(a) *Term "observational study" used in the title.*<br><br>(b) *Summary provided in the abstract.*	RECORD 1.1: The type of data used should be specified in the title or abstract. When possible, the name of the databases used should be included.<br><br>RECORD 1.2: If applicable, the geographic region and timeframe within which the study took place should be reported in the title or abstract.<br><br>RECORD 1.3: If linkage between databases was conducted for the study, this should be clearly stated in the title or abstract.	1.1: *Type of data specified in the title. Name of database included in abstract.*<br><br>1.2: *UK stated in the title; timeframe reported in the abstract.*<br><br>1.3: *Single database used in the study.*
**Introduction**					
Background rationale	2	Explain the scientific background and rationale for the investigation being reported	*Explained in the introduction*		
Objectives	3	State specific objectives, including any prespecified hypotheses.	*Stated in the introduction (final paragraph).*		
**Methods**					
Study Design	4	Present key elements of study design early in the paper	*Described in final paragraph of introduction.*		
Setting	5	Describe the setting, locations, and relevant dates, including periods of recruitment, exposure, follow-up, and data collection	*Described in the methods, sub-section 'Setting'.*		

Participants	6	*(a) Cohort study* - Give the eligibility criteria, and the sources and methods of selection of participants. Describe methods of follow-up<br>*Case-control study* - Give the eligibility criteria, and the sources and methods of case ascertainment and control selection. Give the rationale for the choice of cases and controls<br>*Cross-sectional study* - Give the eligibility criteria, and the sources and methods of selection of participants<br><br>*(b) Cohort study* - For matched studies, give matching criteria and number of exposed and unexposed<br>*Case-control study* - For matched studies, give matching criteria and the number of controls per case	(a) *Described in methods sub-section 'Participants', with further details in the online supplement.*<br><br>(b) *Not relevant. This study did not seek to make comparisons requiring a control group.*	RECORD 6.1: The methods of study population selection (such as codes or algorithms used to identify subjects) should be listed in detail. If this is not possible, an explanation should be provided.<br><br>RECORD 6.2: Any validation studies of the codes or algorithms used to select the population should be referenced. If validation was conducted for this study and not published elsewhere, detailed methods and results should be provided.<br><br>RECORD 6.3: If the study involved linkage of databases, consider use of a flow diagram or other graphical display to demonstrate the data linkage process, including the number of individuals with linked data at each stage.	6.1: *All code lists are included in the online supplement.*<br><br>6.2: *Description of methodological details included in the online supplement.*<br><br>*6.3: CHIA (formerly HHRA) is a single database. During the time of this study, HHRA contained linked routinely collected primary and secondary care data on individual patients and this is explained at the start of the online supplement. Deterministic linkage based on NHS number is carried out within the NHS environment. All data is then patient-anonymised prior to researcher access.*
Variables	7	Clearly define all outcomes, exposures, predictors, potential confounders, and effect modifiers. Give diagnostic criteria, if applicable.	*Defined in methods sub-section 'Participants' and 'Statistical Methods'. Additional details in the online supplement.*	RECORD 7.1: A complete list of codes and algorithms used to classify exposures, outcomes, confounders, and effect modifiers should be provided. If these cannot be reported, an explanation should be provided.	7.1: *A complete code list is provided in the online supplement.*

Data sources/ measurement	8	For each variable of interest, give sources of data and details of methods of assessment (measurement). Describe comparability of assessment methods if there is more than one group	*Sources of data and details are fully described in the online supplement. The group is a single cohort, therefore there is no comparison of assessment methods.*		
Bias	9	Describe any efforts to address potential sources of bias	*The study is a 'real-world' study, using routinely collected electronic health record data from GP practices in Hampshire, UK. Since this COPD cohort is derived from entire practice registers, and since the vast majority of the general population are registered with a GP practice, a code-derived cohort should be virtually free of selection bias. Although biases may arise from the differential coding of patient events, we are unaware of any large systematic biases in relation to the variables we examined.*		
Study size	10	Explain how the study size was arrived at	*Since our study was designed to assess the consistency of airflow obstruction in patients*		

			alive with a COPD diagnosis, our study cohort comprised all living COPD patients (n=14,378) in our chosen data source.		
Quantitative variables	11	Explain how quantitative variables were handled in the analyses. If applicable, describe which groupings were chosen, and why	*For descriptive purposes, summary measures for all quantitative variables are presented (table 1). For categorisation of airflow obstruction (AFO), the accepted cut-off for FEV1/FVC% of <70% was used to define AFO and how the FEV1/FVC% were handled is explained in the Statistical Methods subsection. For the regression analysis, the clinical quantitative variables age and number of comorbidities were modelled as continuous variables, while gender, smoking status and body mass index were treated as categorical variables. Impossible values for clinical variables were censored, as described in the online supplement.*		

Statistical methods	12	(a) Describe all statistical methods, including those used to control for confounding<br>(b) Describe any methods used to examine subgroups and interactions<br>(c) Explain how missing data were addressed<br>(d) *Cohort study* - If applicable, explain how loss to follow-up was addressed<br>*Case-control study* - If applicable, explain how matching of cases and controls was addressed<br>*Cross-sectional study* - If applicable, describe analytical methods taking account of sampling strategy<br>(e) Describe any sensitivity analyses	(a) *Regression methods are described in sub-section entitled* ‘Statistical Methods’*. Adjustment for potential confounding was carried out within a regression modelling framework. For continuous variables, descriptive statistics were used which were appropriate for the distribution of each variable.*<br>(b) *We performed a secondary subgroup analysis of AFO in the subset of patients without concurrent asthma (asthma Read codes recorded during the study period (2011-13.) and this is described in the results section.*<br>(c) *Levels of missing data for spirometry and for baseline demographic and clinical characteristics are shown in table 1.*<br>(d) *The study was of three years duration (2011-2013) and patient records were censored at 31st December 2013. Only living patients with continuous*		

			practice data over these 3 years were included in the cohort (described in participant subsection of methods).<br>(e) *Two sensitivity analyses were performed to define those patients in the cohort whose COPD diagnosis was based solely on (1) codes for chronic bronchitis, or (2) codes for acute exacerbation of COPD (outline method described in statistical methods subsection). Relevant defining codes are listed in the online supplement.*<br>*A 3rd sensitivity analysis explored predictors of absent AFO (vs. variable/persistent AFO) using individual comorbidities, rather than number of comorbidities, in the multivariable regression model (table S1 in supplementary material). A 4th sensitivity analysis explored predictors of variable AFO (vs. persistent AFO), in which an "asthma ever" diagnosis was a*		

			separate covariate in the model (the number of comorbidities in this analysis was taken as the number of comorbidities minus asthma).		
Data access and cleaning methods		..		RECORD 12.1: Authors should describe the extent to which the investigators had access to the database population used to create the study population.<br><br>RECORD 12.2: Authors should provide information on the data cleaning methods used in the study.	12.1: *Described in 'Data Handling' section of the online supplement.*<br><br>12.2: *Described in 'Data Handling' section of the online supplement.*
Linkage		..		RECORD 12.3: State whether the study included person-level, institutional-level, or other data linkage across two or more databases. The methods of linkage and methods of linkage quality evaluation should be provided.	12.3: *Person-level linkage of primary and secondary care data exists within the NHS environment (see 6.3), but this study used only data recorded in primary care.*
**Results**					
Participants	13	(a) Report the numbers of individuals at each stage of the study (*e.g.*, numbers potentially eligible, examined for eligibility, confirmed eligible, included in the study, completing follow-up, and analysed)<br>(b) Give reasons for non-participation at each stage.<br>(c) Consider use of a flow diagram	(a) *Number of eligible patients reported in table 1.*<br><br>(b) *Not applicable for this study.*<br>(c) *Not required.*	RECORD 13.1: Describe in detail the selection of the persons included in the study (*i.e.*, study population selection) including filtering based on data quality, data availability and linkage. The selection of included persons can be described in the text and/or by means of the study flow diagram.	13.1: *Described in methods, sub-section 'Participants'. All living patients aged over 25 years with a primary care diagnosis of COPD and continuous follow-up data were included in the study cohort (14,378 patients). Their data are shown in tables 1,3,5 and 6 of the main article and in the supplementary table S1. Of the 12,491 patients with recorded FEV1/FVC*

					ratios, 11,331 had values observed for all variables in the multivariable regression model for absent AFO vs. persistent/variable AFO, all of which were included in the regression analysis reported in table 2 of the main article and in the supplementary table S1. 10,105 patients had values observed for all variables used in the multivariable regression model for variable AFO vs. persistent AFO, all of which were included in the regression analysis reported in table 4 of the main article.
Descriptive data	14	(a) Give characteristics of study participants (*e.g.*, demographic, clinical, social) and information on exposures and potential confounders<br>(b) Indicate the number of participants with missing data for each variable of interest<br>(c) *Cohort study* - summarise follow-up time (*e.g.*, average and total amount)	(a) *Provided in table 1*<br><br>(b) *Provided in table 1).*<br><br>(c) *The cohort consisted of patients with continuous data and alive at the end of the study (31/12/13), as described in participant subsection of methods. Spirometry values (used to categorise patients by AFO) comprised all values of FEV1/FVC% in each patient's*		

			records from their initial COPD diagnosis until 31/12/13.		
Outcome data	15	*Cohort study* - Report numbers of outcome events or summary measures over time<br>*Case-control study* - Report numbers in each exposure category, or summary measures of exposure<br>*Cross-sectional study* - Report numbers of outcome events or summary measures	*Summary measures of respiratory prescribing over the 3-year period are reported in tables 5 and 6.*		
Main results	16	(a) Give unadjusted estimates and, if applicable, confounder-adjusted estimates and their precision (e.g., 95% confidence interval). Make clear which confounders were adjusted for and why they were included<br><br>(b) Report category boundaries when continuous variables were categorized	(a) *Adjusted estimates and 95% confidence intervals are provided in tables 2 and 4 of the main article and in the supplementary table S1 for multivariable logistic regression analyses. All measured variables were included in the regression models. In tables 2 and 4, the model used the <u>number</u> of comorbidities as covariate, while the supplementary table S1 shows multivariable regression estimates using individual comorbidities as covariates.*<br>(b) *Category boundaries for FEV1/FVC% are described in statistical*		

		(c) If relevant, consider translating estimates of relative risk into absolute risk for a meaningful time period	*methods and reported in tables 1 and 3. Category boundaries for BMI are shown in table 2*<br>(c) *not applicable*		
Other analyses	17	Report other analyses done—e.g., analyses of subgroups and interactions, and sensitivity analyses	*An analysis of inhaled medication during the 3 years for the subgroup without concurrent asthma codes is reported in table 6. A summary of the sensitivity analyses (AFO in patients whose defining codes were solely based on (1) chronic bronchitis codes, or (2) acute exacerbation of COPD codes) is reported in the final text of the results section. The sensitivity analyses using multivariable logistic regression to estimate the odds of absent AFO and individual comorbidities is shown in supplementary table S1.The results of the sensitivity analyses using multivariable logistic regression to estimate the odds of variable AFO, in which "asthma ever" was a separate covariate, are*		

			summarised as text in the manuscript results.		
**Discussion**					
Key results	18	Summarise key results with reference to study objectives	*Summarised in the early paragraphs of the discussion.*		
Limitations	19	Discuss limitations of the study, taking into account sources of potential bias or imprecision. Discuss both direction and magnitude of any potential bias	*Presented in the discussion; study limitations are considered and results put in context with other evidence.*	RECORD 19.1: Discuss the implications of using data that were not created or collected to answer the specific research question(s). Include discussion of misclassification bias, unmeasured confounding, missing data, and changing eligibility over time, as they pertain to the study being reported.	19.1: *Implications of using routine data are addressed in the discussion.*
Interpretation	20	Give a cautious overall interpretation of results considering objectives, limitations, multiplicity of analyses, results from similar studies, and other relevant evidence	*Presented in the discussion; study limitations are considered and results put in context with other evidence.*		
Generalisability	21	Discuss the generalisability (external validity) of the study results	*Addressed in the last paragraph of the discussion. Further details relating to generalisability of CHIA data, including indices to measure socioeconomic deprivation, described on page 1 of the online supplement.*		
**Other Information**					
Funding	22	Give the source of funding and the role of the funders for the present study and, if applicable, for the original study on which the present article is based	*Provided in the support statement and at the beginning of the methods section.*		
Accessibility of protocol, raw data,		..		RECORD 22.1: Authors should provide information on how to access any	22.1: *A detailed description of the*

and programming code				supplemental information such as the study protocol, raw data, or programming code.	*database, data handling, code methodology and all code lists are provided in the online supplement to which the reader is directed from the main body of the text.*

*Reference: Benchimol EI, Smeeth L, Guttmann A, Harron K, Moher D, Petersen I, Sørensen HT, von Elm E, Langan SM, the RECORD Working Committee. The REporting of studies Conducted using Observational Routinely-collected health Data (RECORD) Statement. *PLoS Medicine* 2015. **12**(10): e1001885

*Checklist is protected under Creative Commons Attribution (CC BY) license.
